# Robot-Assisted Radical Cystectomy with Intracorporeal Urinary Diversion: Perioperative Management and Operative Technique

**DOI:** 10.3390/jcm15207858

**Published:** 2026-10-12

**Authors:** Cristofer Zillo, Ashti Shah, Agustin Perez-Londono, Jinlei Li, Zyad J. Carr, Fady Ghali, Wei Shen Tan

**Affiliations:** 1 Department of Urology, Yale School of Medicine, 789 Howard Avenue, New Haven, CT 06520, USA; 2 Department of Anesthesia, Yale School of Medicine, 333 Cedar Street, P.O. Box 208051, New Haven, CT 06520, USA; 3 Pre-Surgical Evaluation Clinic, Yale New Haven Hospital, 20 York Street, New Haven, CT 06510, USA; 4 Division of Surgery and Interventional Science, Charles Bell House, 43–45 Foley Street, London W1W 7TS, UK

**Keywords:** bladder cancer, radical cystectomy, robotic surgery, urinary diversion, ileal conduit, orthotopic neobladder, enhanced recovery

## Abstract

Radical cystectomy with bilateral pelvic lymphadenectomy and urinary diversion remains the standard of care for muscle-invasive bladder cancer and selected high-risk non-muscle-invasive disease. The procedure carries substantial morbidity, in part because the typical patient is elderly and burdened by cardiorespiratory comorbidity. Robot-assisted radical cystectomy (RARC) was developed to attenuate this morbidity, and RARC with intracorporeal urinary diversion (iRARC) avoids the laparotomy that earlier randomized trials required for extracorporeal reconstruction. In this technical narrative review, we present a single-institution operative approach to iRARC, distinguishing institutional practice from evidence-based recommendations. We describe patient selection, preoperative optimization and enhanced recovery, and provide a step-by-step account of our operative technique for male and female radical cystectomy, pelvic lymph node dissection template, and totally intracorporeal ileal conduit and orthotopic neobladder reconstruction. We also summarize the perioperative, oncological, and functional outcomes of iRARC, which support its role as a safe option with specific perioperative advantages in experienced, high-volume centers.

## 1. Introduction

Radical cystectomy, bilateral pelvic lymphadenectomy, and urinary diversion remain the standard-of-care treatment for muscle-invasive bladder cancer (MIBC) and selected high-risk non-muscle-invasive bladder cancer (NMIBC) [1]. The patients requiring this operation are often elderly and have significant cardiovascular and respiratory comorbidities attributable to long-standing tobacco exposure. This technically demanding procedure inevitably carries a significant risk of morbidity and mortality, with historical open series reporting 90-day complication rates exceeding 50% and perioperative mortality of up to 3% [2].

Efforts to minimize morbidity prompted the development of minimally invasive radical cystectomy. Although the first laparoscopic cystectomy was described in 1992, the approach was never widely adopted because intracorporeal urinary diversion proved technically prohibitive [3]. The robotic platform overcame many of these constraints, and robot-assisted radical cystectomy (RARC) has been increasingly adopted, particularly in high-volume centers.

An important distinction has shaped the evidence base. The early randomized trials comparing RARC with open radical cystectomy (ORC), including RAZOR and the Memorial Sloan Kettering trial, reconstructed the urinary diversion extracorporeally through a laparotomy incision [4,5]. Conversion to an open diversion arguably masks the benefit of a truly minimally invasive operation. Our own meta-analysis of these extracorporeal trials found no perioperative advantage over ORC beyond reduced blood loss [6]. A subsequent important step has been the transition to RARC with totally intracorporeal urinary diversion (iRARC), in which both the cystectomy and the diversion are completed robotically. The iROC trial, which randomized patients to iRARC or ORC, showed that a completely intracorporeal operation modestly increased the number of days alive and out of hospital within 90 days (median 82 vs. 80 days) and lowered the risk of thromboembolic and wound complications, although the investigators noted that the clinical importance of the primary end point difference remains uncertain [7]. Retrospective institutional data further suggest that iRARC may act synergistically with an enhanced recovery after surgery (ERAS) pathway to reduce both complications and length of stay [8].

It has been proposed that the benefits of the robotic platform are realized when the diversion is performed intracorporeally. Avoiding a laparotomy that an extracorporeal diversion requires removes a source of wound morbidity and postoperative pain, which in turn lessens opioid requirements and may hasten the return of bowel function. These mechanisms may contribute to the lower perioperative morbidity observed in randomized comparisons [7,9].

In this technical narrative review, we describe the operative approach developed at our institution for iRARC. We outline patient selection, preoperative optimization, the operative technique for male and female radical cystectomy, the pelvic lymph node dissection template, and totally intracorporeal ileal conduit and orthotopic neobladder reconstruction, before summarizing the enhanced recovery pathway and the perioperative, oncological, and functional outcomes that underpin contemporary practice.

### Article Type, Terminology, and Literature Selection

This article is a technical narrative review that combines a description of our institutional operative technique and perioperative pathway with a narrative synthesis of the supporting literature. The relevant literature was identified through a non-systematic search of PubMed/MEDLINE for English-language articles published up to August 2026, using combinations of the terms “robot-assisted radical cystectomy”, “intracorporeal urinary diversion”, “ileal conduit”, “orthotopic neobladder”, “open radical cystectomy”, “enhanced recovery”, “pelvic lymph node dissection”, “nerve-sparing”, “organ-sparing”, and “perioperative systemic therapy”. Priority was given to randomized controlled trials, meta-analyses of randomized trials, international guidelines, and consensus statements; large observational series and technical descriptions were included where higher-level evidence was lacking, and additional articles were identified from reference lists. Institutional preferences are identified as such in the text and are distinguished from recommendations supported by comparative evidence; Table 1 summarizes the evidence underpinning the principal elements described. Throughout, RARC refers to robot-assisted radical cystectomy irrespective of the diversion technique, and iRARC refers specifically to RARC with totally intracorporeal urinary diversion.

## 2. Patient Selection

Patient selection for iRARC broadly mirrors that of open cystectomy. Contraindications are few but include inability to tolerate pneumoperitoneum or prolonged steep Trendelenburg positioning because of severe cardiopulmonary disease or raised intracranial or intraocular pressure. The Pasadena consensus advises that, while still on the learning curve, surgeons should consider deferring cases involving morbid obesity, previous pelvic irradiation, bulky locally advanced disease, or previous pelvic surgery, in which adhesions and distorted planes heighten the risk of rectal or vascular injury intraoperatively. It is suggested that at least 20–30 procedures are needed to flatten the initial learning curve [31].

### 2.1. Physiologically High-Risk Patients

iRARC may be of particular value in patients with physiological compromise. In institutional cohort studies, we observed that patients with limited cardiorespiratory reserve and patients with preoperative anemia undergoing iRARC did not experience the adverse perioperative outcomes that have been associated with these risk factors in open surgery [24,25]. The minimal blood loss and reduced fluid shifts of a totally intracorporeal operation may benefit those patients at greatest risk of complications after laparotomy; however, these observations have not been confirmed in randomized trials.

### 2.2. Mitigating the Disadvantages of the Robotic Approach

The principal disadvantage of iRARC is the requirement for a prolonged operation in steep Trendelenburg, which raises ventilatory and intraocular pressures. However, physiological studies confirm that hemodynamic and pulmonary variables remain within safe limits and are well tolerated [35], and operative time falls with experience. Nonetheless, in patients with obesity, raised intraocular pressure, fixed lower-limb deformity, or hip osteoarthritis, our practice is to minimize head-down tilt by breaking the operating table at the mid-abdominal point and side-docking the robot, which allows the lower limbs to lie in a near-horizontal position while preserving pelvic access. In cases where steep Trendelenburg is required, the degree of tilt can be reduced during the construction of the urinary diversion, and when possible, the patient should be supine rather than in lithotomy to reduce the risk of compartment syndrome from prolonged pressure on the calves.

### 2.3. Perioperative Systemic Therapy and Implications for Surgical Planning

Perioperative systemic therapy should be considered for patients with MIBC before cystectomy, with the regimen determined by cisplatin eligibility, comorbidity, patient preference, and regional availability. Neoadjuvant cisplatin-based chemotherapy has long been the standard for cisplatin-eligible patients, with a higher pathological complete response rate than cystectomy alone (38% vs. 15%) and an absolute 5-year overall survival benefit of 5% [12,13]. The addition of perioperative durvalumab to neoadjuvant gemcitabine–cisplatin improved event-free and overall survival in the NIAGARA trial (24-month overall survival 82.2% vs. 75.2%) [16]. More recently, perioperative enfortumab vedotin (EV) plus pembrolizumab has been evaluated in two distinct populations. In KEYNOTE-905/EV-303, which enrolled patients who were ineligible for or declined cisplatin, EV–pembrolizumab improved 2-year overall survival compared with cystectomy alone (79.7% vs. 63.1%; absolute difference 16.6%) [14]. In KEYNOTE-B15/EV-304, which enrolled cisplatin-eligible patients, EV–pembrolizumab improved 2-year overall survival compared with neoadjuvant gemcitabine–cisplatin (86.9% vs. 81.3%; absolute difference 5.6%) [15]. For patients with high-risk pathology after cystectomy, adjuvant nivolumab improved disease-free survival [17]. In patients with molecular residual disease after cystectomy, circulating tumor DNA (ctDNA)-guided adjuvant atezolizumab improved disease-free and overall survival [18]. Treatment selection therefore remains individualized and should be agreed upon within a multidisciplinary team.

These regimens carry substantial toxicity: grade ≥ 3 adverse events of any cause occurred in 71.3% of patients receiving EV–pembrolizumab in KEYNOTE-905/EV-303 (vs. 45.9% with cystectomy alone) and in 75.7% in KEYNOTE-B15/EV-304 (vs. 67.2% with gemcitabine–cisplatin) [14,15]. Surgical planning should therefore account for treatment-related toxicity and for the adjuvant component of these regimens, which depends on timely recovery after cystectomy. In our institution, cystectomy is performed 4–5 weeks after completion of neoadjuvant treatment. Whether the faster early recovery associated with iRARC facilitates the timely delivery of, or improves tolerance to, adjuvant therapy—for example, in patients with residual disease or detectable ctDNA [18,38]—has not been evaluated; this should be regarded as a hypothesis for prospective study rather than an established advantage of the robotic approach.

## 3. Preoperative Optimization

### 3.1. Preoperative Planning

We perform staging CT imaging within two months of radical cystectomy to confirm the absence of interval metastatic disease. Male patients being considered for a nerve-sparing or prostate-capsule-sparing cystectomy should have a prostate-specific antigen (PSA) test and a prostate MRI to exclude the presence of clinically significant prostate cancer. When a neobladder is planned, a prior prostatic urethral biopsy is preferred, although not mandatory. In our practice, a frozen section of the distal prostatic urethra to exclude urothelial disease determines whether we proceed with neobladder reconstruction. Additionally, bladder neck disease is not an absolute contraindication for neobladder urinary diversion [29]. Prostate MRI may also be used in patients undergoing neobladder diversion to assess membranous urethral length, which may inform continence counseling by extrapolation from radical prostatectomy data [33]. We do not perform routine prostate biopsy, reserving it for cases in which imaging or PSA raises suspicion of prostate cancer. Pelvic MRI is also useful for surgical planning in patients with bladder cancer involving the rectum or vagina.

Female organ-sparing cystectomy is not a standard approach, but it may be discussed with carefully selected women in whom oncological safety is not compromised; supporting evidence is limited to small, predominantly retrospective series with a high risk of bias [30]. In our practice, sexually active patients with organ-confined (≤cT2 N0) tumors away from the trigone and bladder neck may be considered for vaginal-sparing cystectomy. Ovarian preservation should be considered, particularly in premenopausal patients, and increasing evidence also supports preservation in postmenopausal women, in whom ovarian androgens are converted to estrogen that is known to be cardioprotective [39]. However, women at increased risk of ovarian cancer, such as those with Lynch syndrome or a BRCA mutation, should undergo bilateral salpingo-oophorectomy at the time of radical cystectomy, based on guidance from gynecological surgery [39]. In patients receiving a neobladder, preservation of the uterus may improve pelvic floor support and continence and has been associated with a lower risk of urinary retention [30,40].

Patients with comorbidities who are deemed high risk for surgery attend the Yale pre-surgical evaluation clinic. Radical cystectomy candidates are often older, frail, and medically complex, with cardiovascular or pulmonary disease, diabetes, chronic kidney disease, obesity, malnutrition, and a high incidence of tobacco exposure. Prior systemic therapy, limited physiological reserve, cognitive vulnerability, and poor social support further increase the likelihood of postoperative complications, prolonged hospitalization, loss of independence, and delayed recovery. Clinic assessment therefore extends beyond conventional history and examination to define modifiable risk and establish an individualized perioperative plan. In select patients, brief submaximal cardiopulmonary exercise testing provides an objective measure of integrated cardiopulmonary reserve, identifying limitations that may be missed by patient-reported functional capacity and supporting informed counseling, postoperative monitoring, and appropriate resource allocation [41]. When time before cystectomy permits, multimodal prehabilitation combines aerobic and resistance exercise, inspiratory muscle training, nutritional and protein support, and psychological preparation. This approach can improve functional reserve and accelerate postoperative recovery, although definitive evidence of a reduction in complications is still evolving [21]. Comprehensive optimization includes medication reconciliation, anemia and iron-deficiency treatment, glycemic and blood pressure control, smoking cessation, pulmonary therapy, nutritional supplementation, delirium prevention, and coordination with anesthesia, geriatrics, cardiology, and primary care. Ultimately, optimal outcomes following radical cystectomy depend on coordinated multidisciplinary care that integrates surgical, anesthetic, medical, nursing, and rehabilitative expertise across the perioperative continuum.

### 3.2. Bowel Preparation, Carbohydrate Loading, and Stoma Siting

In our practice, patients consume a low-residue diet for two days before surgery, which clears the ileal segment of solid content that is otherwise difficult to evacuate by endoscopic suction. Solid food is permitted until six hours before surgery, and clear fluids until two hours before surgery. Routine mechanical bowel preparation is unnecessary; however, in our practice, we administer an early-morning enema to empty the rectum in patients with a history of pelvic radiation, who are at higher risk of rectal injury. High-calorie carbohydrate drinks are recommended the evening before surgery to prevent a catabolic state and the insulin resistance associated with the surgical stress response. For a planned ileal conduit, the stoma site is marked preoperatively by the stoma care nurse in both the sitting and standing positions.

### 3.3. Anesthetic and Pharmacological Optimization

Our institutional protocol for pre-emptive analgesia comprises single-shot spinal anesthesia with 100–200 µg of preservative-free morphine, together with single-injection, bilateral, ultrasound-guided anterior quadratus lumborum blocks using a weight-based dose of long-acting local anesthetic (bupivacaine or ropivacaine) with the adjuvants dexamethasone sodium phosphate 10 mg and methylprednisolone acetate 80 mg. The regional anesthesia techniques are achieved preoperatively under standard American Society of Anesthesiologists (ASA) monitoring and mild intravenous sedation with midazolam and/or fentanyl, with the patient in the left lateral decubitus position, without the need for repositioning. This reduces the postoperative fall in blood pressure and the incidence of motor block. It also frees the patient from a continuous infusion pump (e.g., epidural or peripheral nerve block catheter infusions); together, these advantages promote early mobilization. The combination of intrathecal morphine and a motor-sparingfascial plane block with long-acting local anesthetic and adjuvants provides effective postoperative and post-discharge analgesia with minimal opioid requirements in our experience; however, this regimen has not been compared with alternative analgesic strategies in cystectomy patients, and regional anesthetic practice varies between institutions. We also utilize an arterial line, placed at the beginning of the case, which allows continuous pressure monitoring and access for blood gas analysis. General anesthesia with endotracheal intubation is then induced and maintained according to standard practice.

At the beginning of the case, mechanical thromboprophylaxis with a sequential compression device, along with 5000 units of subcutaneous heparin, is administered. Intravenous broad-spectrum antibiotics covering Gram-negative organisms and anaerobes, typically cefazolin and metronidazole, are given within 60 min of incision and, in our practice, continued for a further 48 h postoperatively. We acknowledge that guidelines generally recommend no more than 24 h of prophylaxis, and a randomized trial in open cystectomy with urinary diversion found 24-h prophylaxis to be non-inferior to extended prophylaxis (until catheters and stents are removed) for surgical site infection [20]; shorter courses are therefore reasonable. After induction, a nasogastric or orogastric tube is placed to deflate the stomach and a urethral catheter to drain the bladder. Ondansetron and dexamethasone are given as antiemetics, and a combination of intravenous acetaminophen and ketorolac is administered for analgesia at the start of the case.

## 4. Surgical Approach

The description below reflects the technique we have refined for totally intracorporeal radical cystectomy and urinary diversion, beginning with the male operation in detail and proceeding to the modifications for female cystectomy and to urinary reconstruction.

### 4.1. Male Radical Cystectomy

#### 4.1.1. Patient Positioning and Port Placement

**Figure 1:**
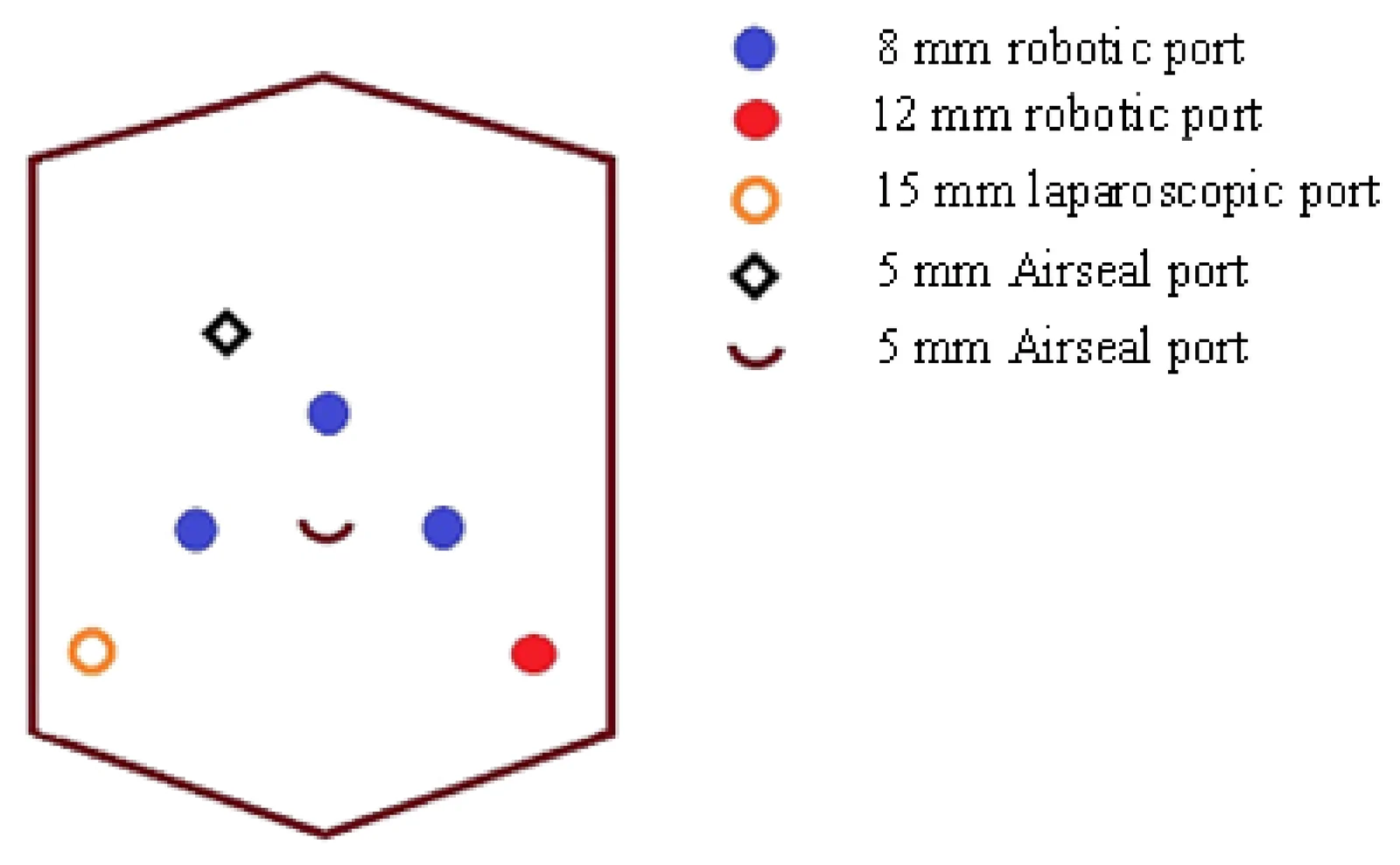
Port configuration for robot-assisted radical cystectomy.

The patient is positioned in 30° supine Trendelenburg on a memory-foam mattress with the arms adducted and tucked to the sides, and all pressure points padded. It is good practice to test the Trendelenburg position before prepping and draping, confirming that the hips lie in a neutral position with slight flexion to avoid pressure injury and femoral neuropraxia. In cases where synchronous urethrectomy is required, the lithotomy position is necessary. A six-port transperitoneal approach is used to enter the peritoneal cavity (Figure 1). After pneumoperitoneum is established with a Veress needle, an 8 mm camera port is introduced at least 4 cm cranial to the umbilicus. Two 8 mm robotic ports are placed just above the level of the umbilicus, lateral to the rectus muscle and a minimum of 8 cm from the camera port. We favor a 15 mm laparoscopic port sited approximately 5 cm above and 1 cm anterior to the right iliac crest, which allows easy removal of lymph node packets using a reusable laparoscopic retrieval bag (Anchor; ConMed, Largo, FL, USA). A 12 mm robotic port is placed in the equivalent position on the left to allow use of a 60 mm robotic stapler (SureForm; Intuitive Surgical, Sunnyvale, CA, USA) for the urinary diversion. Finally, a 5 mm valveless insufflation port (AirSeal; ConMed, Largo, FL, USA) is used to maintain a constant and uniform pneumoperitoneum and is sited cranial to the line joining the camera and the first robotic arm. All ports are placed under direct vision, and a 0° lens is used throughout. The robot is side-docked, and the pneumoperitoneum is reduced to 10 mmHg. Our preferred instruments are monopolar scissors, a Maryland bipolar forceps, and a ProGrasp forceps.

#### 4.1.2. Posterior Dissection

**Figure 2:**
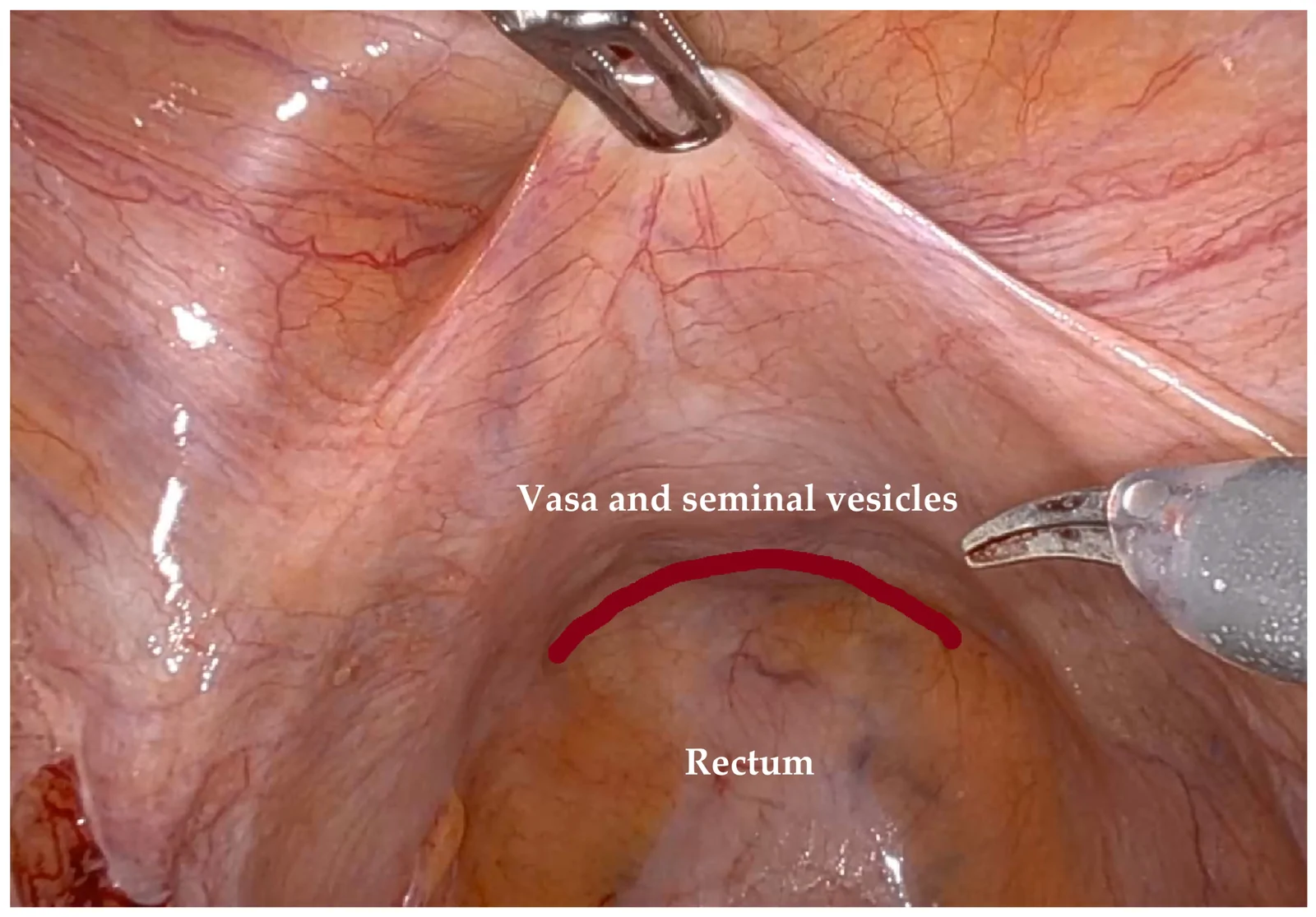
Pelvic anatomy prior to commencing posterior dissection with the ProGrasp forceps retracting the bladder peritoneum anteriorly.

**Figure 3:**
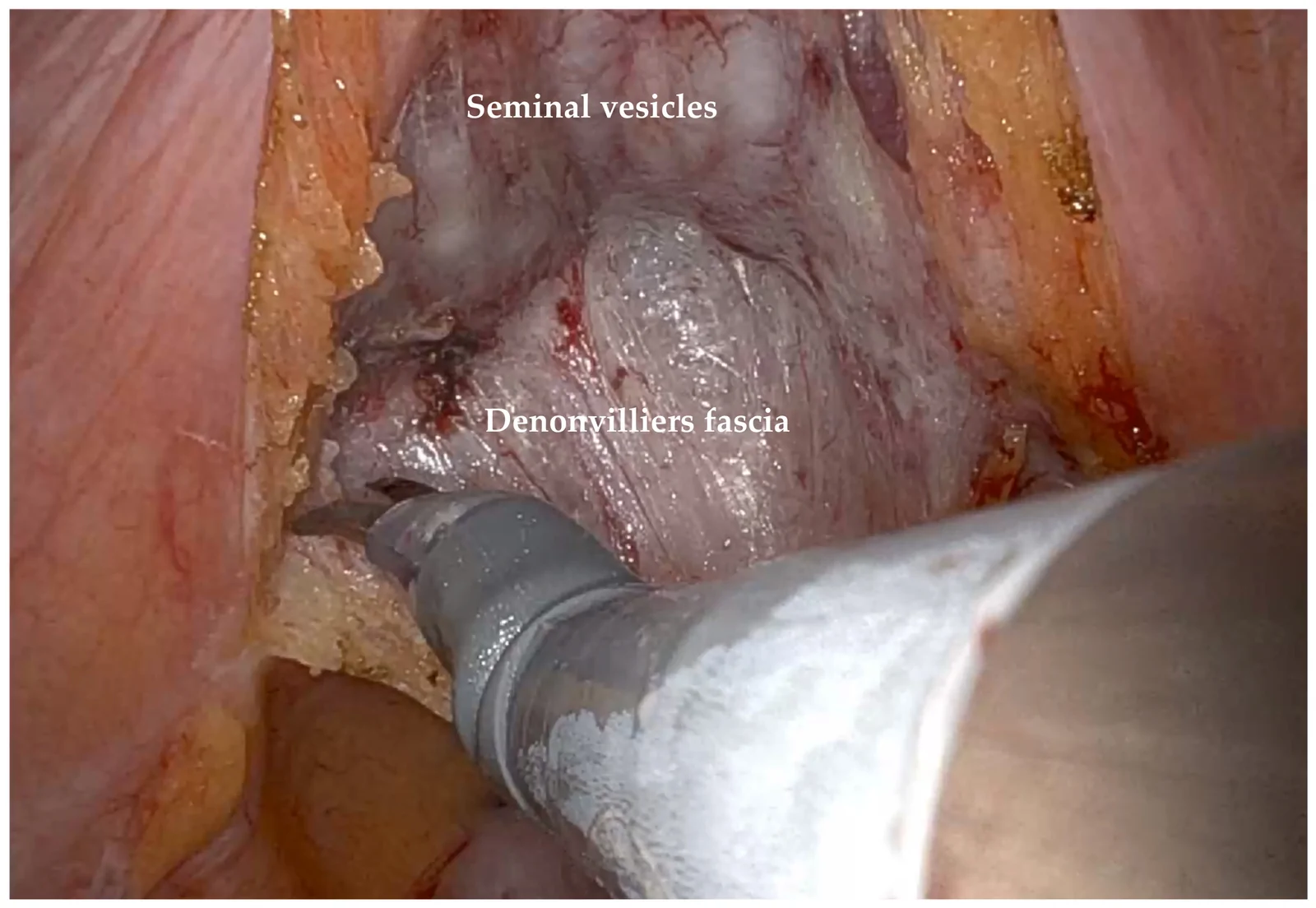
Posterior dissection following incision of the peritoneum.

After the pelvic anatomy is assessed and any adhesions are divided, the peritoneal fold overlying the rectovesical pouch is lifted and a transverse incision is made just distal to where the vasa become prominent as they run medially toward the prostate (Figure 2 and Figure 3). The retrovesical plane is developed within the fatty layer using a combination of blunt dissection and diathermy, with the bladder retracted anteriorly, and the prerectal fat swept toward the rectum. The key landmarks are the rectum with its perirectal fat posteriorly and the seminal vesicles and bladder anteriorly. At the insertion of the seminal vesicles, Denonvilliers’ fascia is incised, and a standard dissection proceeds toward the rectourethralis muscle. When performing a salvage cystectomy, the plane between the bladder, prostate, and rectum is frequently obscured by a desmoplastic layer. In these cases, the dissection should stay anterior to this plane and as close to the prostate as possible to avoid rectal injury.

#### 4.1.3. Nerve-Sparing Technique

**Figure 4:**
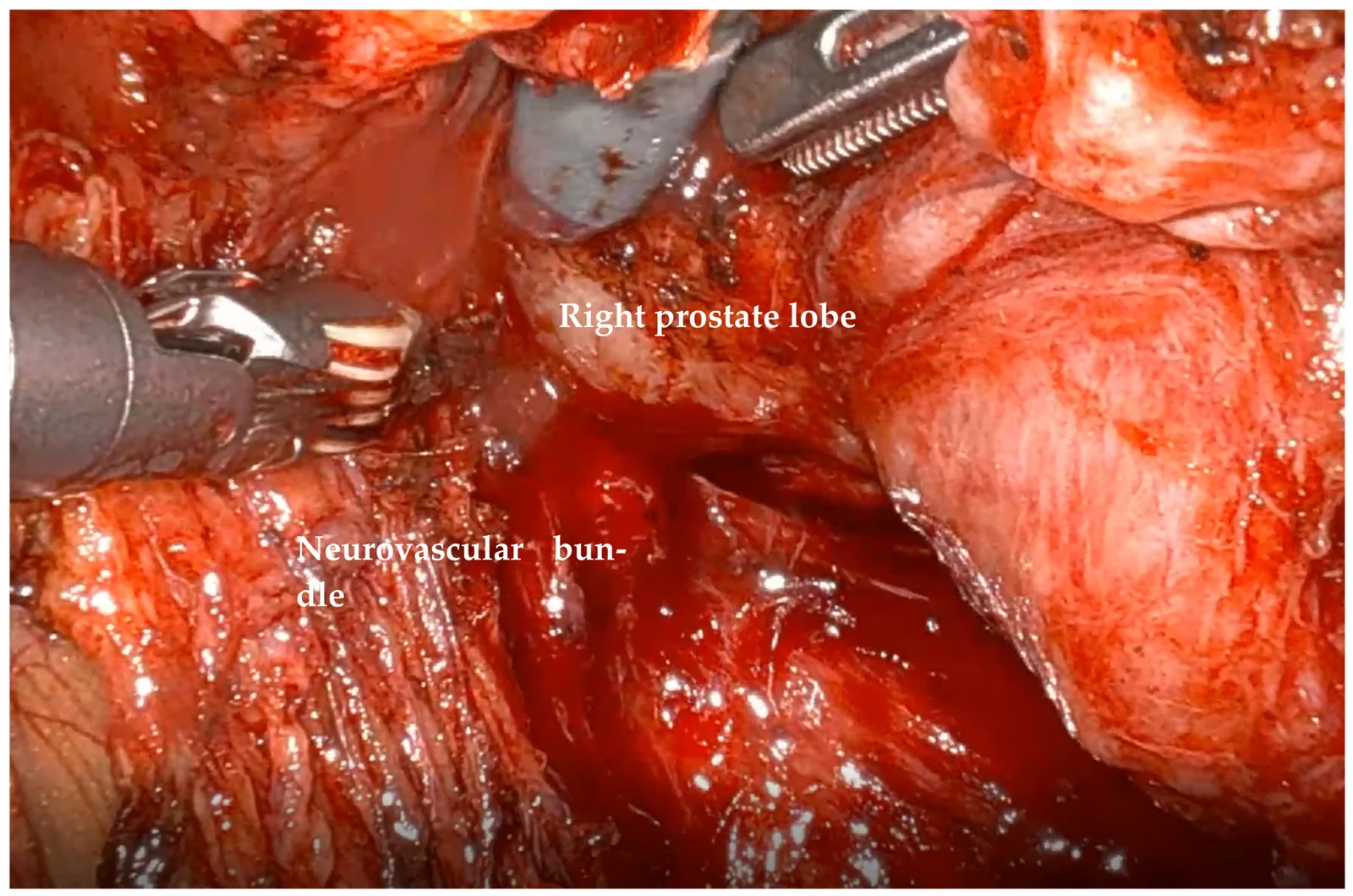
Combination of sharp and blunt dissection with spot coagulation when needed to dissect the neurovascular bundle off the prostate.

Where nerve preservation is intended, defining the intrafascial or interfascial neurovascular plane at the outset of the posterior dissection is crucial. Once the correct plane is entered in the midline, it is developed in an antegrade fashion, preserving the neurovascular bundle that lies posterolateral to the prostate (Figure 4). Hemostasis is typically achieved using bipolar energy or spot monopolar diathermy; in a non-randomized radical prostatectomy series, judicious use of energy was not associated with inferior erectile outcomes compared with athermal dissection [34], although comparable data in cystectomy are lacking.

#### 4.1.4. Prostate Capsule Sparing

**Figure 5:**
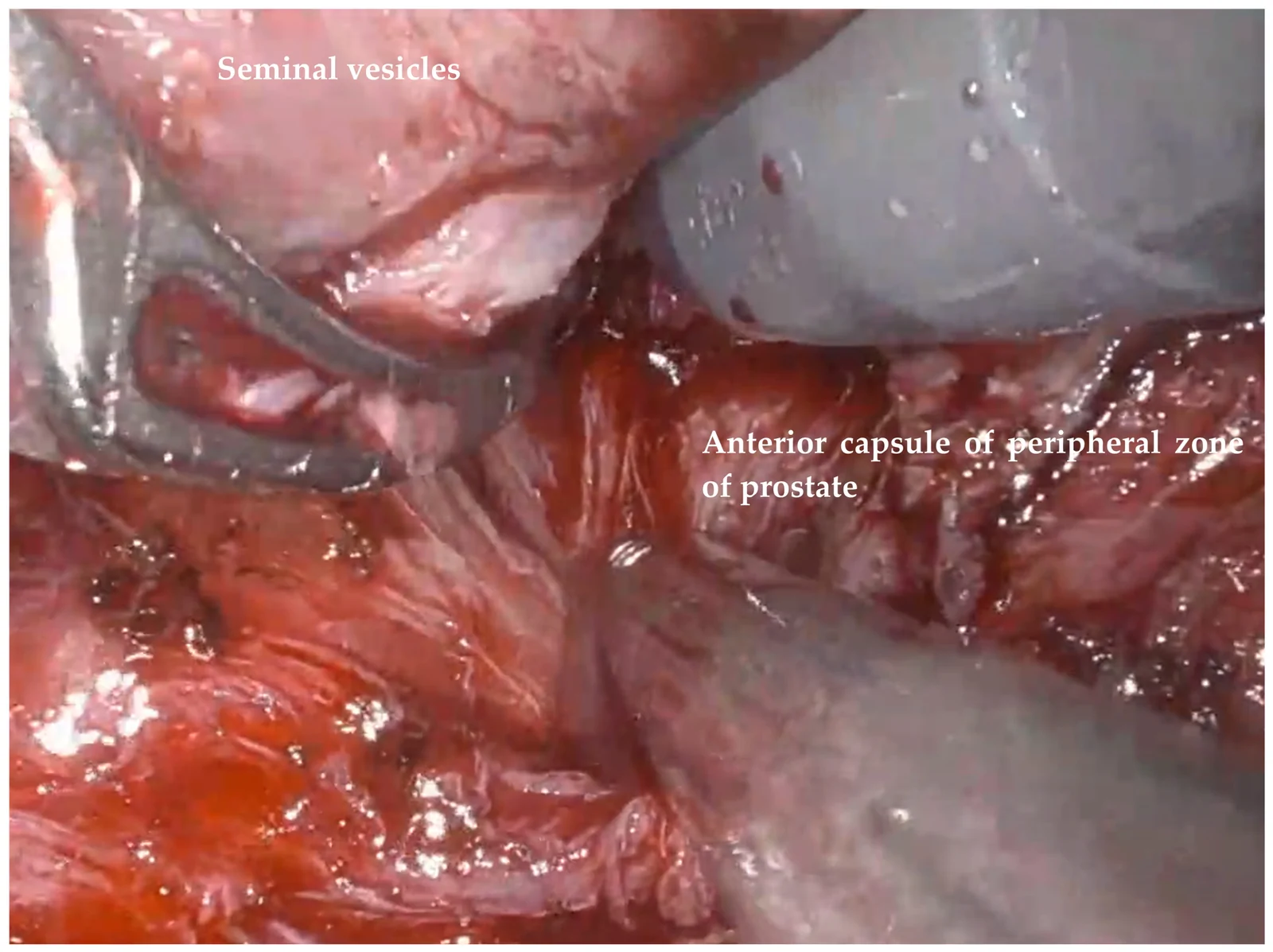
Posterior dissection developing the plane between the central and peripheral zones of the prostate.

A prostate-capsule-sparing variation may be offered to highly selected younger patients who place a high value on potency and continence. Oncological selection requires not only confident exclusion of clinically significant prostate cancer but also exclusion of urothelial carcinoma involving the prostatic urethra in patients with organ-confined disease without extensive carcinoma in situ [29]. Patients should be counseled that this remains a non-standard approach, supported by non-randomized series with a moderate-to-high risk of bias, and that the retained prostatic tissue requires ongoing surveillance [29]. The prostate is incised with monopolar energy at its base to enter the plane between the peripheral zone and the central and transitional zones (Figure 5). This achieves complete preservation of the neurovascular bundles, which travel lateral and posterior to the peripheral zone. Avoiding dissection around the seminal vesicles maximizes preservation of the erectile nerves. This approach should not be undertaken when there is any concern for prostatic adenocarcinoma or urothelial involvement of the prostate.

#### 4.1.5. Mobilization of the Ureters and Lateral Dissection

Our preference is to begin the lateral dissection on the left side. Mobilization of the descending and sigmoid colon along the white line of Toldt facilitates exposure of the ureter. The medial umbilical ligament is retracted medially, and the vas deferens is identified, clipped with polymer ligating clips (Hem-o-lok; Teleflex, Morrisville, NC, USA) at its proximal and distal ends, and divided. The distal vas and the medial umbilical ligament are then retracted medially to open the lateral perivesical space, which is developed within the fat toward the endopelvic fascia; the fascia is then opened.

**Figure 6:**
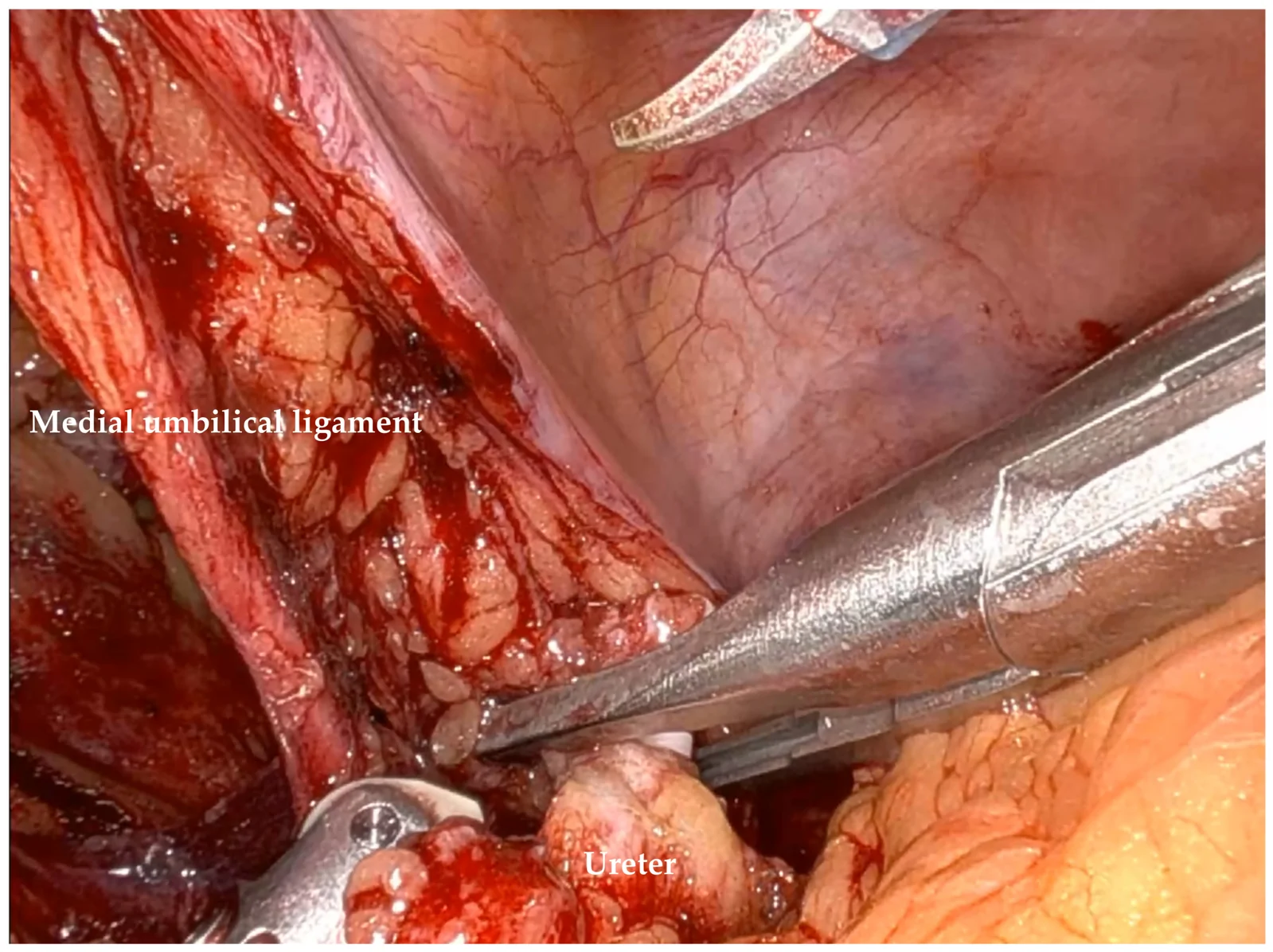
Dissection and identification of the ureter, which is located medial to the medial umbilical ligament.

The left ureter is identified where it crosses the common iliac vessels and travels down the pelvis just medial to the medial umbilical ligament (Figure 6). The periureteric tissue is preserved to maintain vascularity, the ureter is never grasped directly, and traction is minimized, all of which reduce the risk of subsequent ureteric stricture; the distal ureter derives its blood supply laterally, so that medial dissection remains relatively avascular. The distal ureter is divided between polymer clips with a 15 cm 3-0 polyglactin stay suture preloaded onto the proximal clip to allow easy identification of the ureter later during reconstruction.

#### 4.1.6. Bladder and Prostatic Pedicle Dissection

**Figure 7:**
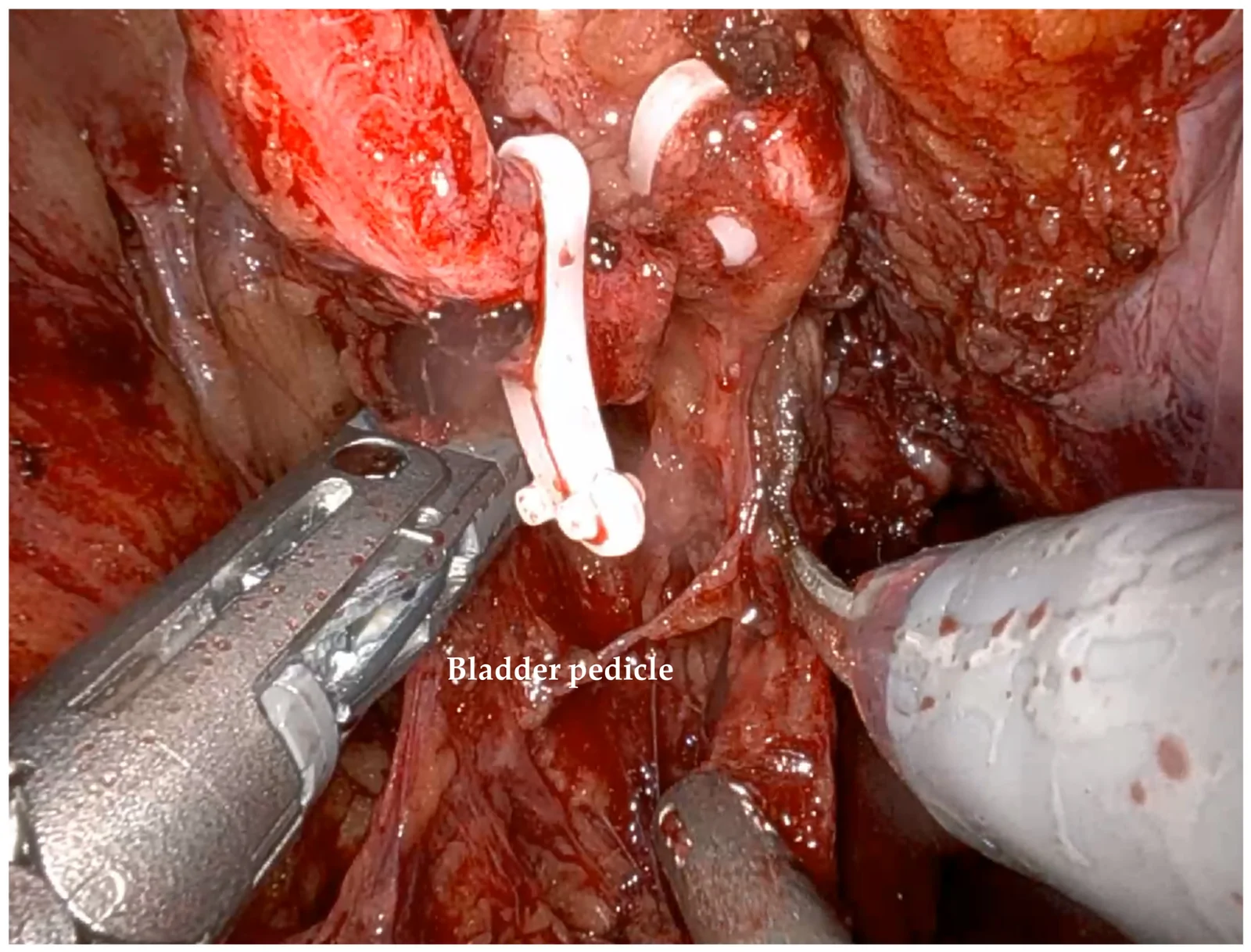
Bladder and prostate pedicles are controlled using a bipolar vessel sealer.

The bladder is retracted medially and upward to hold the lateral pedicle almost perpendicular to the pelvic side wall. This exposes, from cranial to caudal, the medial umbilical ligament, the superior and inferior vesical pedicles, and the prostatic pedicle. It is essential to confirm the position of the divided distal ureter, which lies medial to the medial umbilical ligament, before the pedicles are divided using a bipolar vessel sealer (Vessel Sealer Extend; Intuitive Surgical, Sunnyvale, CA, USA) or between polymer clips (Figure 7). For a nerve-sparing operation, following hemostatic control of the bladder pedicles, monopolar scissors and a Maryland bipolar forceps are used to dissect the prostatic pedicle and neurovascular bundle in a manner analogous to nerve-sparing radical prostatectomy.

#### 4.1.7. Apical Dissection

**Figure 8:**
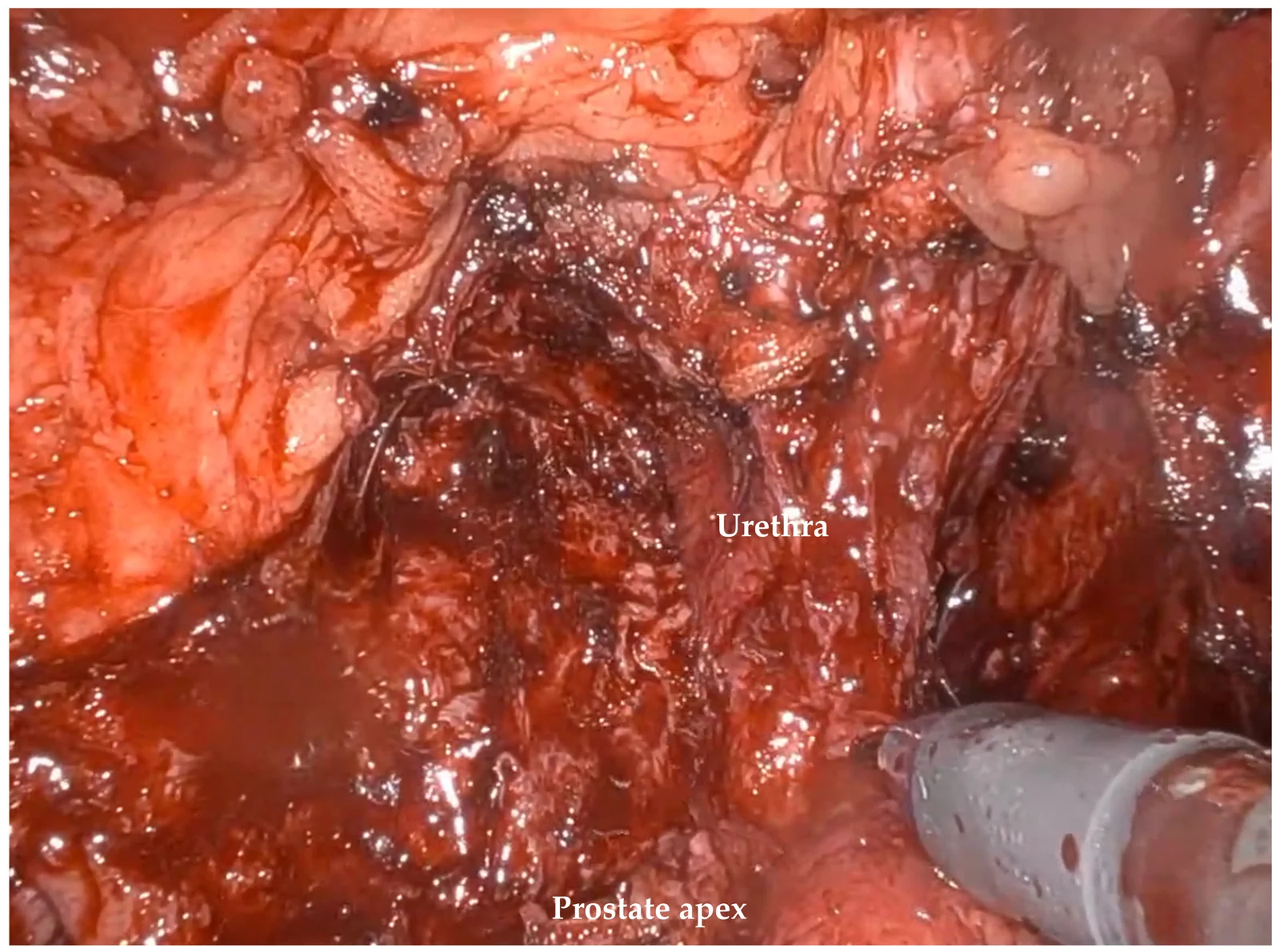
Apical dissection to maximize urethral length to promote urinary continence in patients undergoing neobladder formation.

The anterior bladder is dropped by dividing the median umbilical ligament through the space of Retzius toward the prostatic apex. The dorsal venous complex is divided with electrocautery for an ileal conduit, or cold-cut for a neobladder to preserve maximal urethral length. Where a neobladder is planned and both prostate cancer and urothelial involvement of the prostatic urethra have been excluded, preserving the prostatic apex and a long segment of membranous urethra is intended to optimize the sphincteric mechanism and continence, a rationale largely extrapolated from radical prostatectomy (Figure 8) [33]. Conversely, where disease involves the trigone or prostatic urethra, a long urethral segment is excised en bloc with the specimen. Where ileal conduit diversion is planned, the membranous urethra is dissected out, the catheter is removed, and two extra-large polymer clips are placed as distally as possible before the urethra is divided to prevent spillage of bladder contents. The freed specimen is placed in a large specimen retrieval bag (Endo Catch; Covidien, Dublin, Ireland) and set aside; the dorsal venous complex is then oversewn with a 2-0 barbed suture for hemostasis. The retained urethral stump and its clip are covered by approximating the pelvic musculature, which minimizes the risk of bowel injury during proximal membranous urethral dissection should a subsequent completion urethrectomy be required.

### 4.2. Modifications for Female Cystectomy

The principles of the male operation apply directly to the female pelvis, with modifications related to the gynecological organs and the vagina. The ureteric, lateral, and pedicle dissections proceed as described; the differences lie in the posterior plane and the management of the uterus, adnexa, and vaginal wall.

#### 4.2.1. Standard Anterior Exenteration

**Figure 9:**
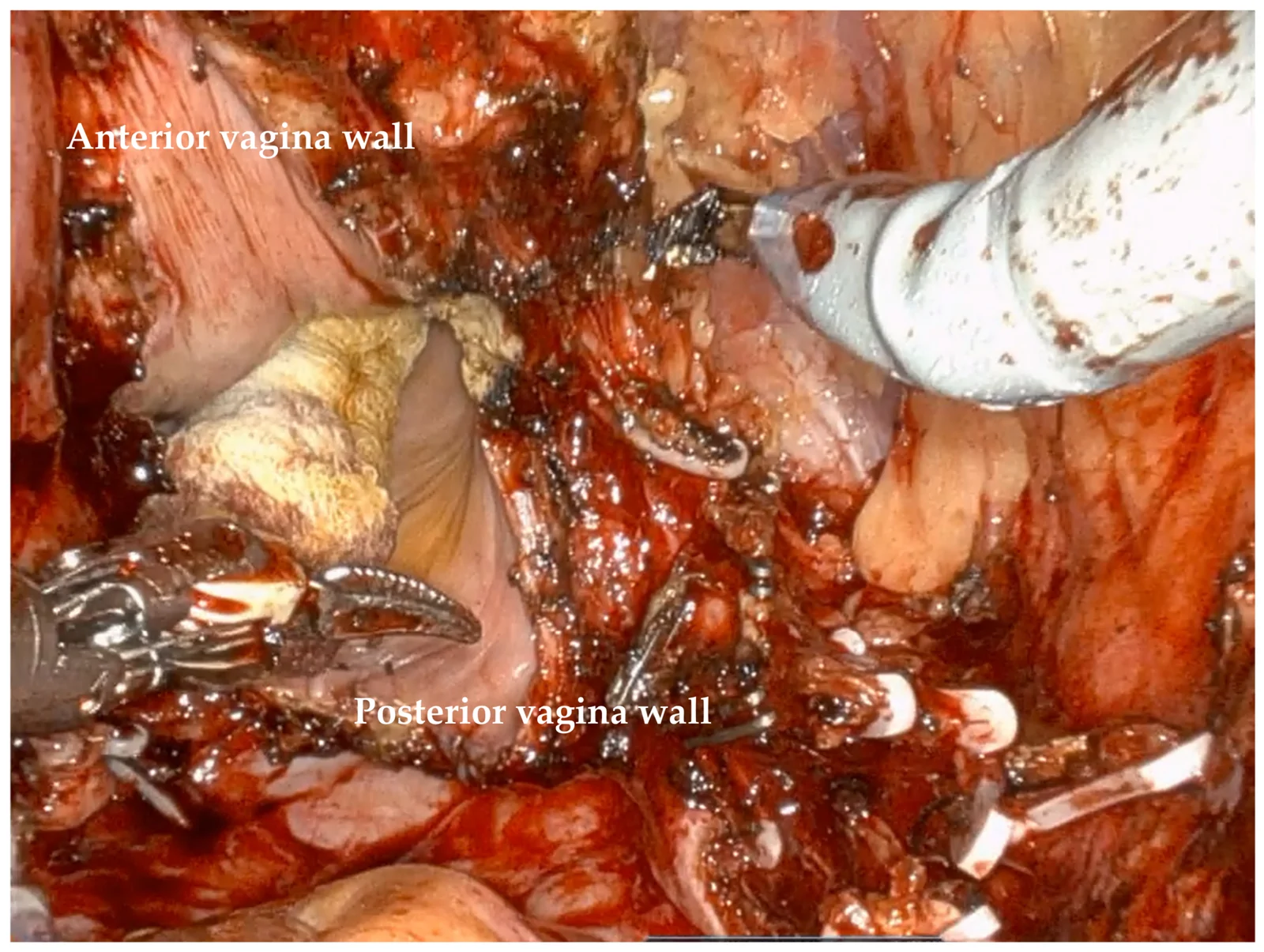
Posterior dissection for a standard anterior exenteration.

In the standard female operation, anterior pelvic exenteration removes the bladder and urethra together with the uterus, adnexa, and anterior vaginal wall. To delineate the posterior plane, a povidone-iodine-soaked swab on a sponge-holder is placed in the posterior vaginal fornix, which renders the fornix readily palpable and visible. The posterior dissection is carried through the pouch of Douglas onto the fornix and continued laterally and caudally along the anterior aspect of the lateral vaginal walls (Figure 9).

The decision to take the urethra en bloc depends on the presence of bladder neck or urethral cancer. Where complete urethrectomy is necessary, as the dissection approaches the introitus, the urethral catheter is passed into the abdomen and retracted cranially to allow the urethral meatus to be circumscribed, and the specimen is removed en bloc. The posterior vagina is freed from the rectum and folded caudally, and the vaginal defect is closed with a 0 absorbable barbed suture.

Where there is no cancer at the bladder neck or urethra, our preference is to dissect the urethra circumferentially as distally as possible. The urethral catheter is removed, and large polymer clips are used to control the urethra and prevent spillage. This preserves the distal anterior vagina, which reduces the risk of vaginal stenosis; facilitates closure of the vaginal defect; and maximizes vaginal length and caliber for sexual function.

#### 4.2.2. Organ-Preserving and Vaginal-Sparing Approaches

Female organ-sparing radical cystectomy includes preservation of one or more of the following structures: the vagina, uterus, fallopian tubes, and ovaries. This non-standard approach should be reserved for highly selected patients who wish to preserve sexual or urinary function and in whom preservation does not compromise the oncological margin. Tumor at the trigone or bladder neck, locally advanced cancer, and any suspicion of vaginal or gynecological involvement are contraindications. Current evidence is limited to small, heterogeneous, largely retrospective series with immature oncological follow-up, and organ preservation should therefore be undertaken only after detailed counseling [30].

Preservation of the ovaries in premenopausal women should be prioritized. In postmenopausal women, there is increasing evidence that the ovaries continue to secrete androgen, which is converted by peripheral aromatization in adipose tissue to estrone, maintaining a low baseline of endogenous estrogen. In a large cohort of women undergoing hysterectomy for benign disease, ovarian conservation was associated with lower cardiovascular and all-cause mortality than bilateral oophorectomy [42]. For women at population risk undergoing hysterectomy without oophorectomy, the American College of Obstetricians and Gynecologists recommends discussing opportunistic salpingectomy, because many epithelial ovarian cancers are thought to arise in the fallopian tube, but notes that the benefit of ovarian conservation diminishes with age [39]. These data derive from gynecological surgery and have not been evaluated in the radical cystectomy setting.

**Figure 10:**
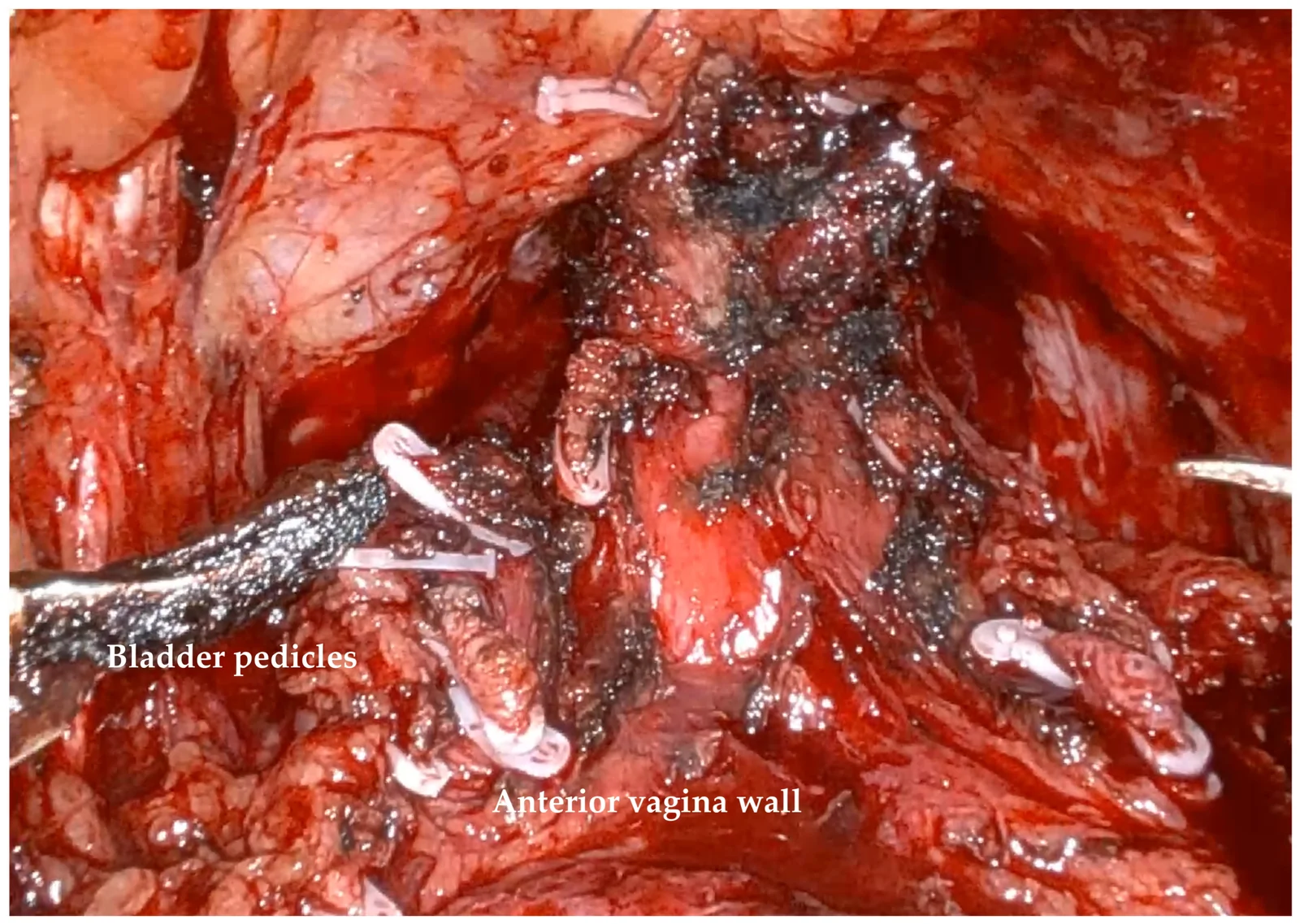
Vaginal-sparing radical cystectomy. Polymer clips were used to control bladder pedicles.

In patients who are candidates for neobladder formation, preservation of the uterus and the anterior vaginal wall is thought to maintain the autonomic innervation and structural support of the pelvic floor that contribute to continence and to the mechanical support of the neobladder. Sparing the anterior vaginal wall in particular avoids the posterior defect that can predispose to a neobladder–vaginal fistula (Figure 10).

### 4.3. Lymph Node Dissection Template

We perform a standard pelvic lymph node dissection comprising the obturator, external iliac, and internal iliac packets. The boundaries are the genitofemoral nerve overlying the psoas laterally, the ureteric crossing of the common iliac artery proximally, the circumflex iliac vein and the node of Cloquet distally and anteriorly, and the hypogastric vessels posteromedially. An extended template is not recommended, given that neither the randomized German AUO AB 25/02 trial nor SWOG S1011 demonstrated a survival benefit for extended over standard lymph node dissection [10,11]. Moreover, extended dissection was associated with a higher rate of major adverse events (54% vs. 44%) and higher 90-day mortality (7% vs. 2%) than the standard template [11].

Dissection is commenced over the lateral border of the external iliac artery and carried to the bifurcation of the common iliac artery using judicious monopolar or bipolar diathermy. Care is taken to protect the genitofemoral nerve lateral to the external iliac artery and the pelvic communicating vessels that enter the psoas lateral to the nodal packet. A split-and-roll technique is used to dissect the nodal packet off the external iliac vessels, and lateral lymphatics are controlled with polymer clips to reduce the risk of lymphocele. Using the laparoscopic suction cannula, the assistant retracts the external iliac vein to allow complete dissection of the obturator packet. The dissection is continued along the lateral pelvic side wall until the obturator nerve is identified; monopolar energy settings are lowered during this portion, and only spot energy is applied to minimize the risk of an obturator reflex. The obturator packet is then stripped off the nerve and dissected bluntly in a cranial direction to free the entire pelvic lymph node packet.

To minimize the risk of port-site recurrence, each nodal packet is placed within a reusable 10 mm laparoscopic retrieval bag and removed intact through a port. The lymph node fossa is irrigated and inspected for bleeding, and a hemostatic agent is applied where necessary. A bipolar vessel sealer is an alternative to the Maryland bipolar forceps that allows a hemostatic dissection of the lymph node packet.

### 4.4. Intracorporeal Ileal Conduit Formation

The ileal conduit remains the most frequently performed diversion and the most straightforward to reconstruct intracorporeally; the principles described here also underpin the afferent and efferent limbs of more complex reconstructions.

#### 4.4.1. Tunneling of the Left Ureter Under the Sigmoid

The left ureter is freed above the level of the common iliac vessels, directly over the psoas muscle, preserving the periureteric fat. An avascular plane analogous to that used in total mesorectal excision is developed anterior to the common iliac vessels from right to left, with care taken not to shear the mesenteric veins. This mesenteric window should be developed widely enough to allow the left ureter to be transposed beneath the sigmoid mesentery, so that both ureters reach a single ileal segment without tension or angulation. The thin-walled mesenteric veins are protected, as bleeding here is troublesome to control, and the ureter is handled by its preloaded stay suture on a polymer clip rather than grasped.

#### 4.4.2. Isolation of Ileum and Restoration of Bowel Continuity (Ileal Conduit)

**Figure 11:**
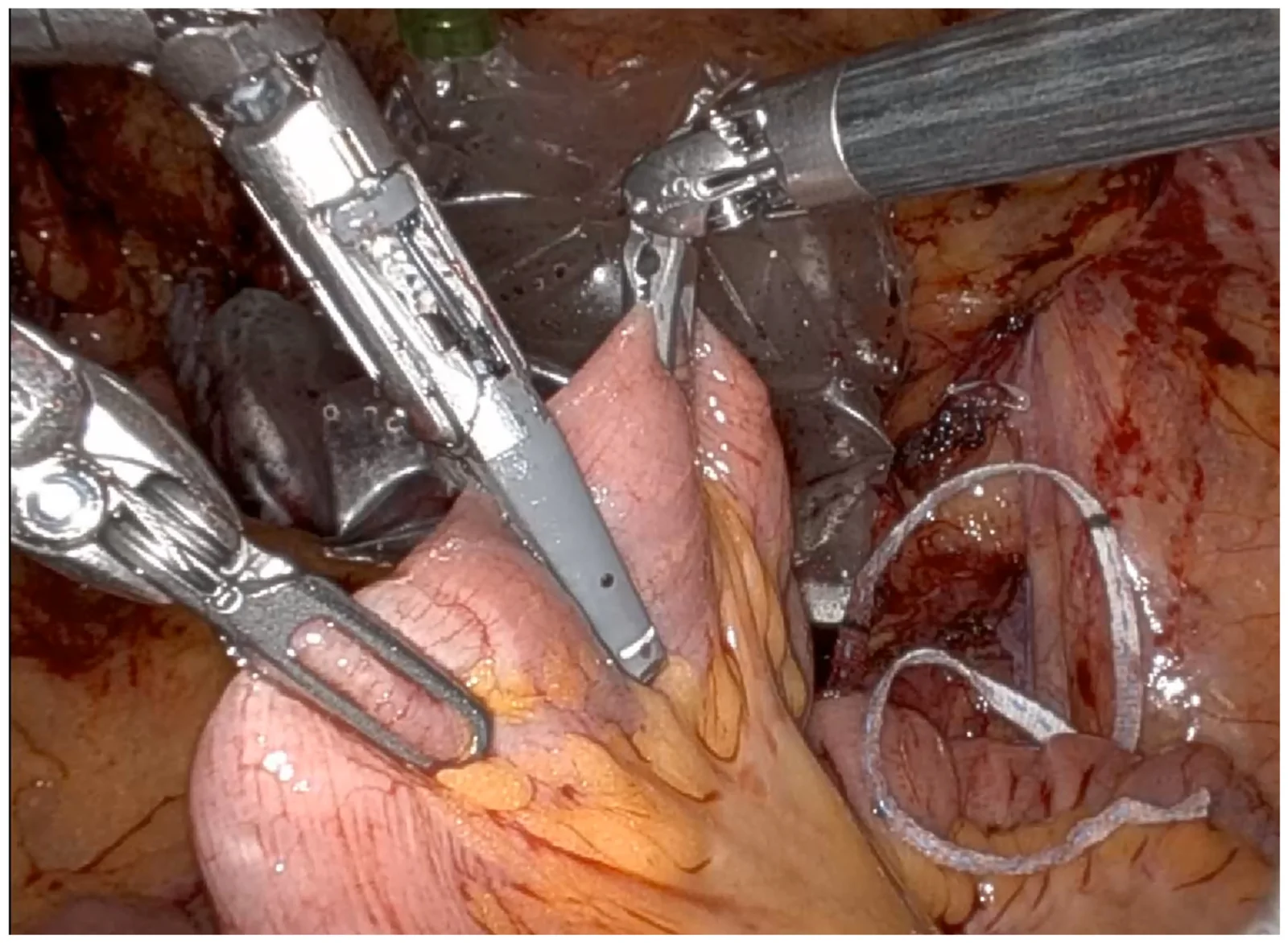
Cadiere forceps are used to lift the ileum to allow the vessel sealer to isolate the bowel segment for ileal conduit formation.

A 15–20 cm segment of distal ileum is selected approximately 15 cm proximal to the ileocecal valve, preserving the terminal ileum and its absorptive function; an umbilical tape aids measurement of the segment to be isolated. Two Cadiere forceps elevate the bowel by its antimesenteric border while a bipolar vessel sealer in the left lateral 12 mm robotic port divides the bowel wall at right angles and then seals and divides the mesentery (Figure 11). After the distal end of the conduit has been divided, the assistant presses on the abdominal wall at the preplanned stoma site. The distal end of the loop of ileum is lifted toward this site, and the proximal end of the conduit can be estimated by noting where the loop of ileum meets the mesenteric window already developed beneath the sigmoid. The proximal end is divided with the vessel sealer in similar fashion, and a 60 mm robotic stapler is fired to seal off the proximal end of the conduit; this allows easy orientation of the conduit and avoids the need for stay sutures, which improves surgical efficiency. Intravenous indocyanine green (ICG), 2.5 mg, can aid assessment of bowel perfusion before restoration of bowel continuity.

Bowel continuity is then restored using two 60 mm robotic stapler loads to create a large-caliber side-to-side antimesenteric anastomosis, with the enterotomy closed using one or two further 60 mm robotic stapler loads. The ileo-ileal anastomosis is positioned cranial to the conduit, and the mesenteric window is closed to prevent internal herniation.

#### 4.4.3. Spatulation and Uretero-Ileal Anastomosis

**Figure 12:**
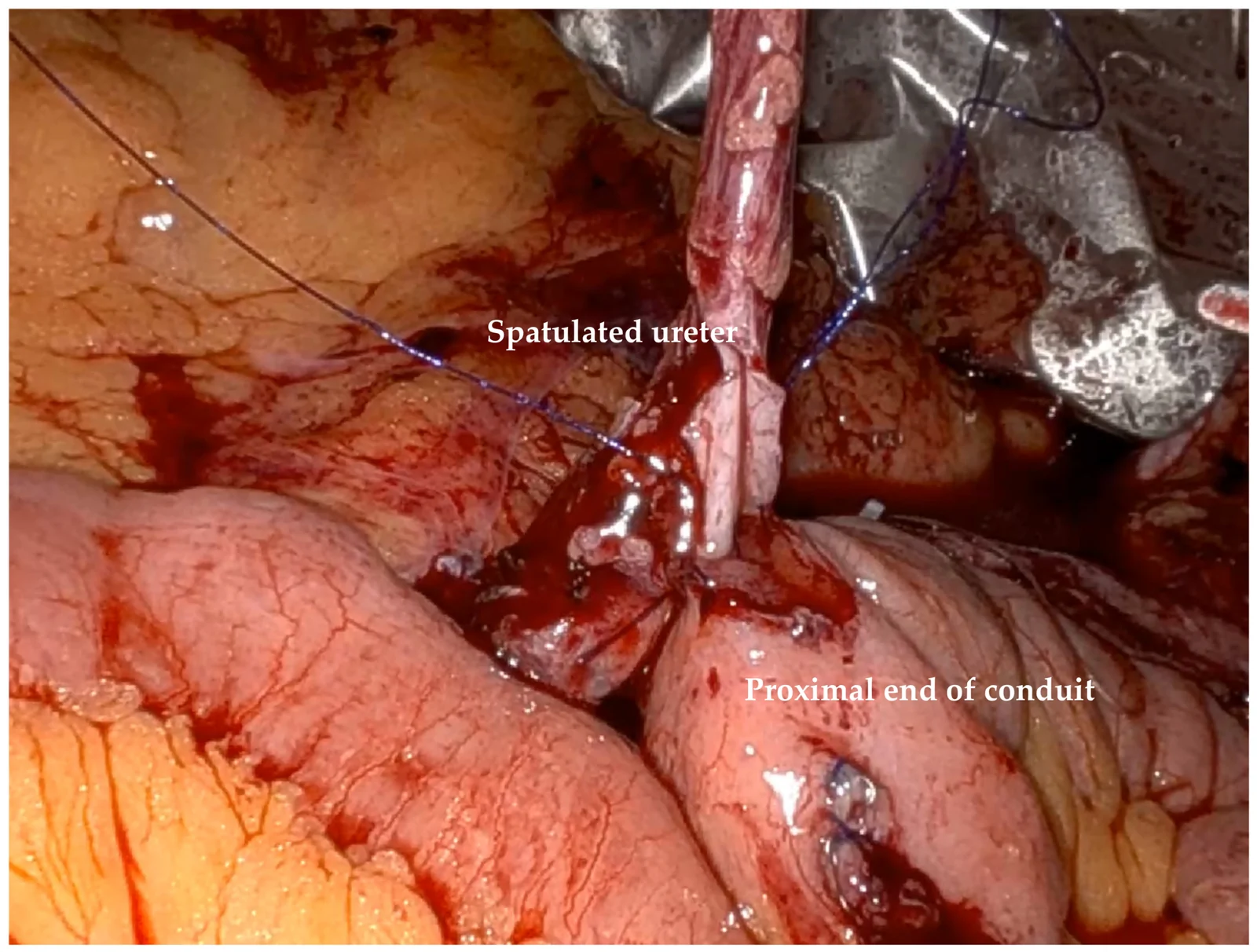
Left distal ureter is suspended anteriorly to aid uretero-ileal anastomosis.

We favor a longer conduit, on the rationale that a shorter ureteric length may reduce the risk of uretero-ileal anastomotic stricture. Intravenous ICG can also be used to assess perfusion of the distal ureters, especially in patients with a history of pelvic radiation. The stay suture on the polymer clip at the distal end of the ureter is held up with a ProGrasp forceps, and the distal ureter is spatulated for approximately 1.5 cm. We favor a Bricker uretero-ileal anastomosis using two continuous 4-0 polydioxanone sutures (Figure 12). Midway through the anastomosis, a 6 Fr single-J stent is passed over a guidewire into the renal pelvis, and its distal end is drawn out through the conduit lumen with a laparoscopic needle driver introduced retrogradely through the conduit. The distal end of the ureter is excised and sent for permanent pathological assessment, and the uretero-ileal anastomosis is completed. Using the same 4-0 polydioxanone suture, the anastomosis is retroperitonealized by closing the overlying peritoneum, which may reduce tension and the risk of urinary leak.

#### 4.4.4. Stoma Formation and Specimen Extraction

The proximal end of the conduit is closed with a 2-0 polyglactin 910 suture, which also secures the stents to prevent slippage. The polyglactin 910 suture is brought to the skin surface using a fascial closure device (Endo Close; Medtronic, Minneapolis, MN, USA) approximately two fingerbreadths below the preplanned site to mature the stoma. The fascial defects at the 12 mm robotic and 15 mm laparoscopic assistant port sites are closed internally with 0 polydioxanone sutures, and a 19 Fr JP drain is placed in the pelvis through the left 8 mm robotic port.

The thumb-plate of a 20 mL syringe is used to mark the skin as a guide for the circumference of the stoma. At the level of the fascia, a cruciate incision measuring approximately 1.5 fingerbreadths is made, and the distal end of the conduit with its stents is delivered using a long Babcock forceps under vision and matured. We favor specimen extraction through a small Pfannenstiel incision to minimize disruption to the conduit and anastomosis during extraction; a supraumbilical midline incision is used for patients receiving a neobladder.

### 4.5. Intracorporeal Neobladder Formation

For patients receiving a neobladder, maximizing urethral length is considered important for continence, a principle extrapolated from radical prostatectomy data [33]. In patients in whom prostate cancer and urothelial involvement of the prostatic urethra have been excluded, the apical prostate can be preserved to provide a long urethral stump. In female patients, the urethra should be divided at the vesicourethral junction to maximize urethral length, with preservation of the distal anterior vaginal wall and endopelvic fascia.

#### 4.5.1. Isolation of Ileum and Restoration of Bowel Continuity (Neobladder)

**Figure 13:**
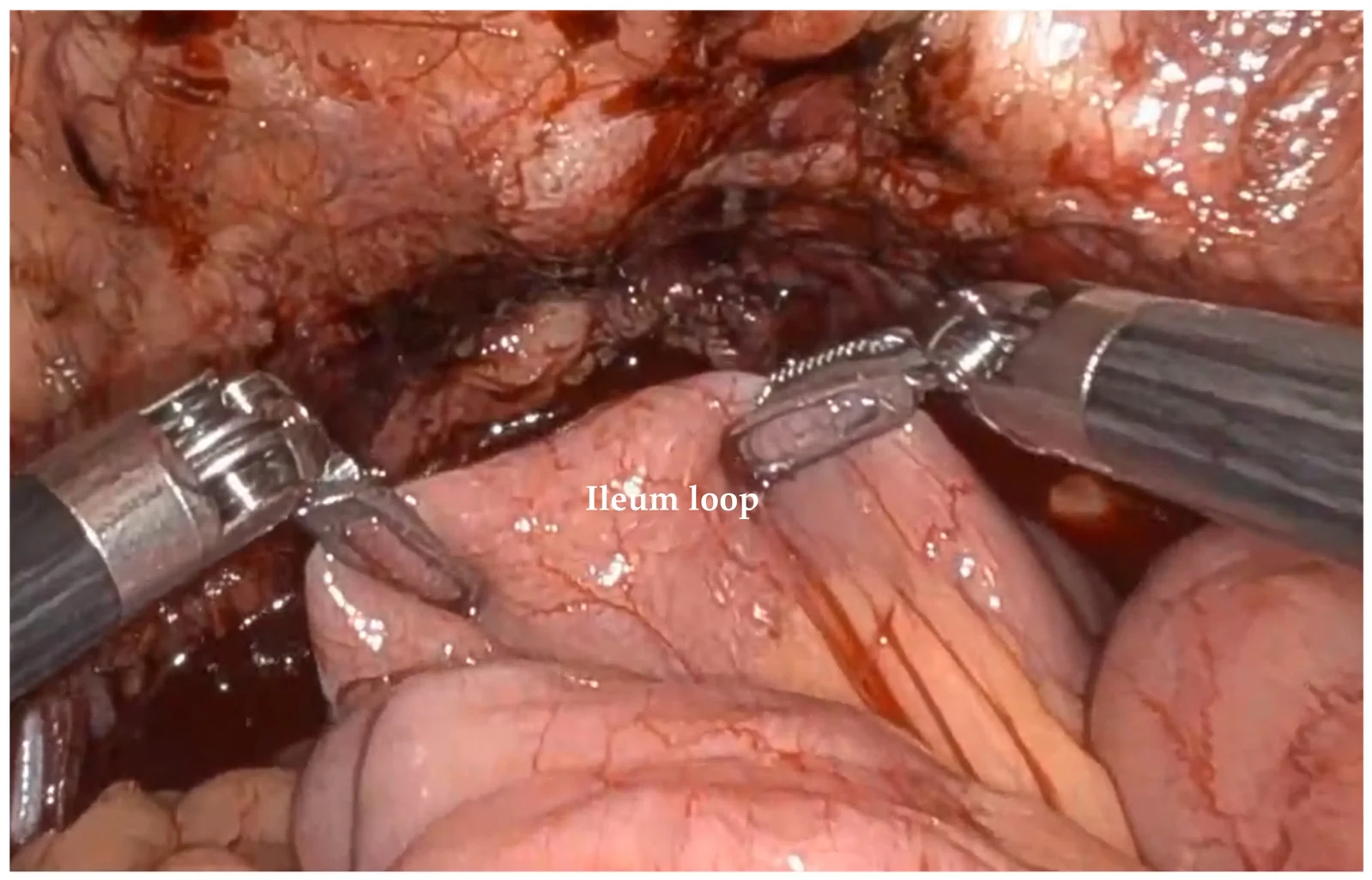
Most dependent loop of ileum is brought down to the deep pelvis.

The most dependent loop of ileum is brought down into the deep pelvis toward the urethra (Figure 13). The Trendelenburg tilt can be reduced to 20° to minimize mesenteric tension, and a small mesenteric incision may aid further mobilization of the bowel toward the urethra. Approximately 10 cm of ileum is measured to the right of the urethra and isolated with a 60 mm robotic stapler using the same approach described in Section 4.4. Following urethro-ileal anastomosis, a further 40 cm to the left of the anastomosis is isolated, and bowel continuity is then restored.

#### 4.5.2. Urethro-Ileal Anastomosis

**Figure 14:**
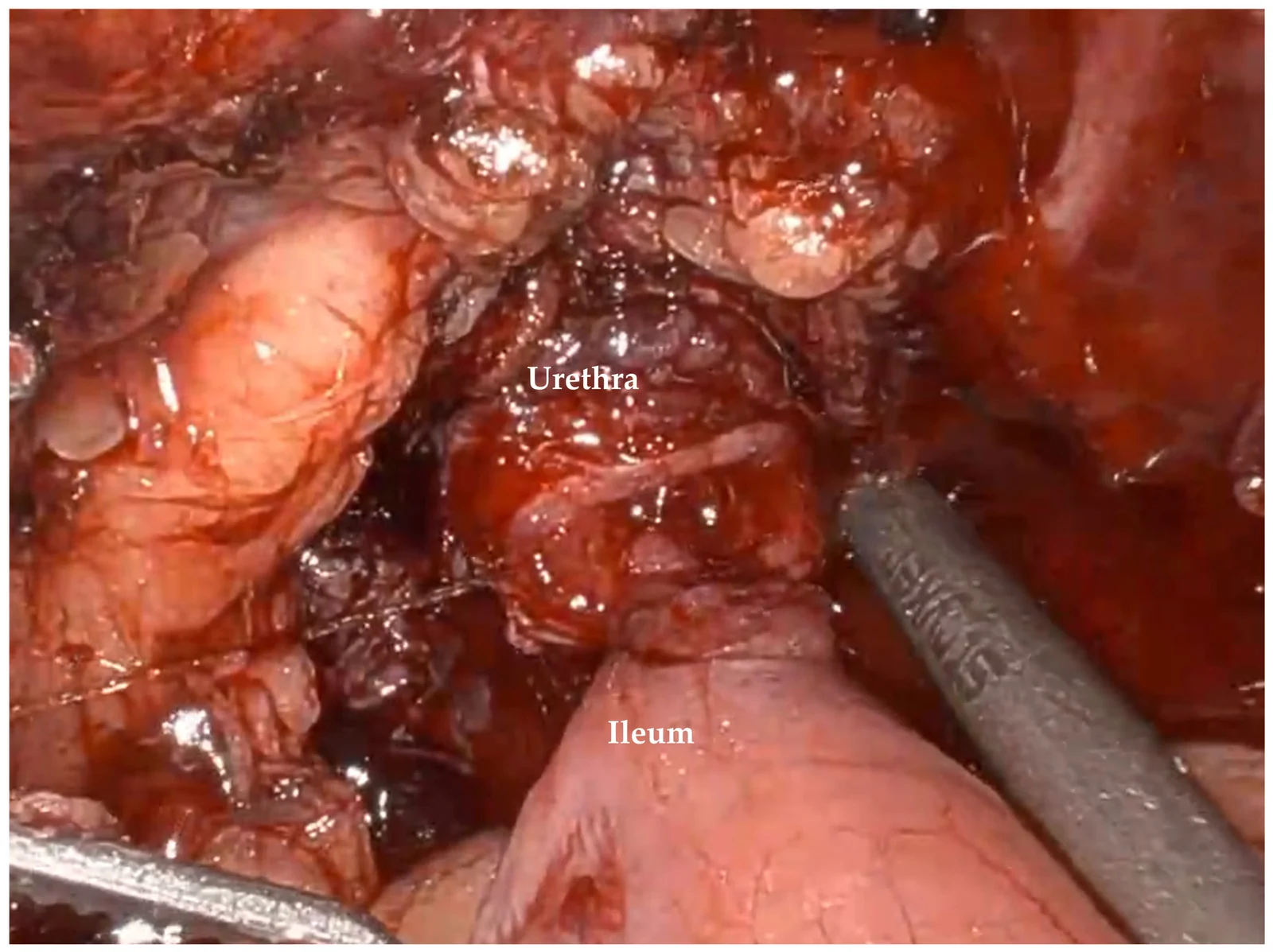
Urethro-ileal anastomosis formation.

A 1 cm incision is made on the antimesenteric side of the bowel at the preplanned urethro-ileal anastomotic site, and the anastomosis is performed with two continuous 3-0 barbed sutures using a modified Van Velthoven technique (Figure 14) [43]. After the first two throws have been placed on each side, at the 5 and 6 o’clock positions, the bowel is parachuted down toward the urethra, and the anastomosis is completed. An 18 Fr Foley catheter is then passed into the bowel lumen and the balloon inflated with 5 mL of water to secure it in position.

#### 4.5.3. Detubularization of Bowel and Neobladder Formation

**Figure 15:**
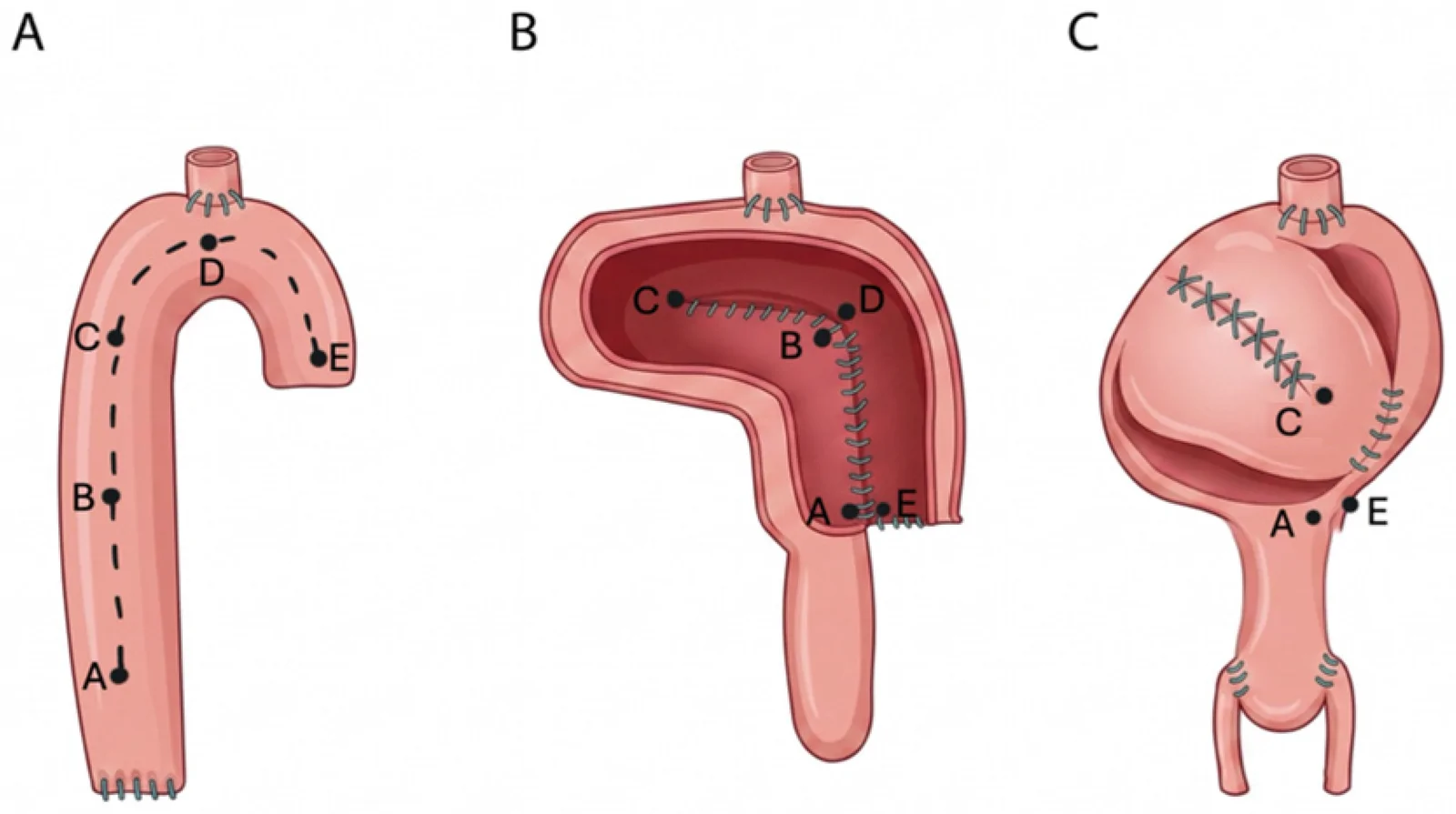
Neobladder formation. Adapted from Lavallée and Wiklund [37]. (**A**) The ileal loop is detubularized from A to E. A to D measures approximately 30 cm; D to E, approximately 10 cm; and A to the staple line, 10 cm. (**B**) Stay sutures are placed between A and E and between B and D on the posterior plate before the posterior neobladder plate is closed with a running barbed suture. (**C**) The neobladder is folded anteriorly from the distal edge toward A and E and sutured closed in a ‘baseball’ configuration. Alternatively, the A–E edge can be folded caudally toward C, depending on the mobility of the mesentery and the bowel length between C and B–D.

**Figure 16:**
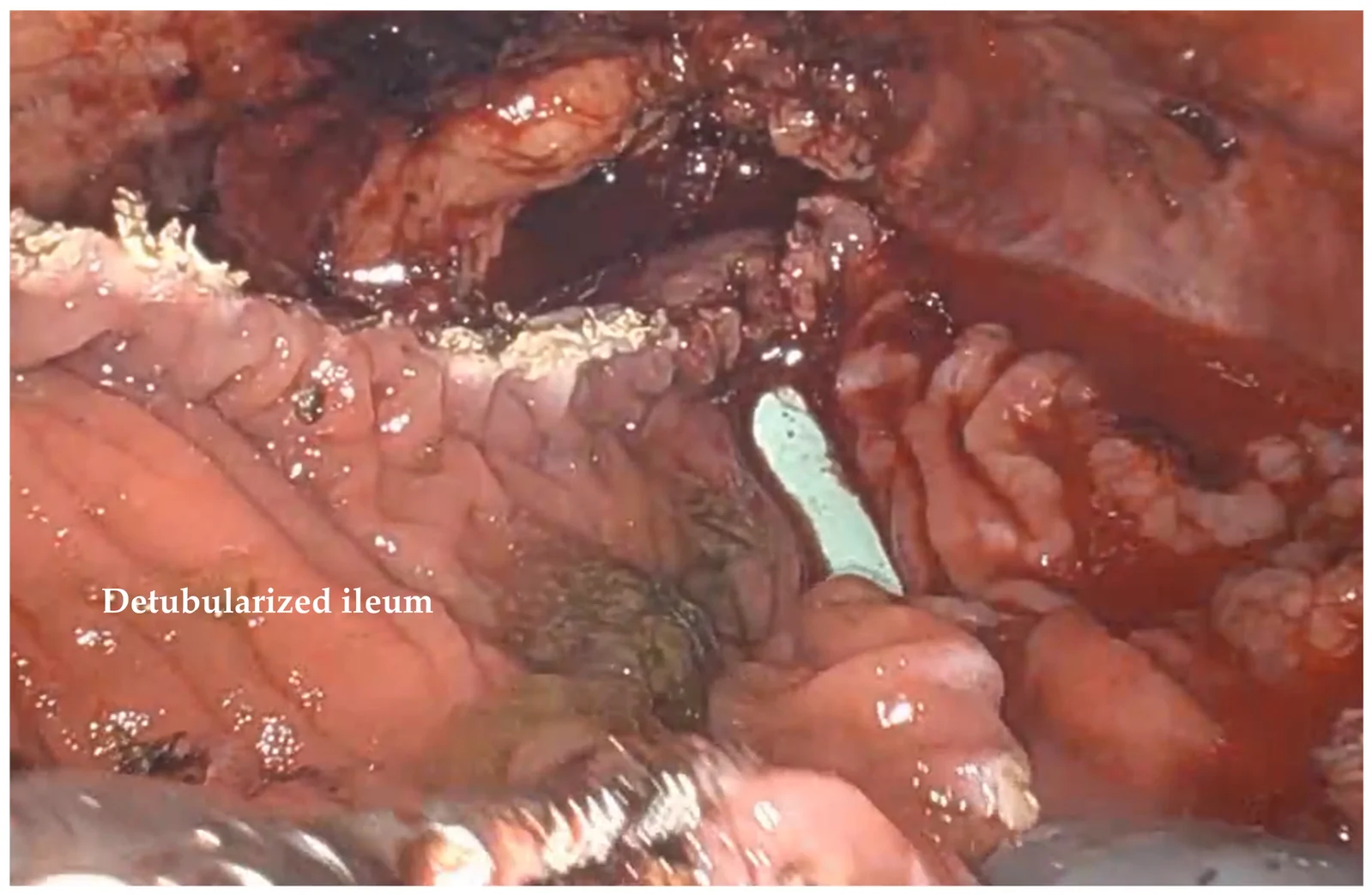
Small bowel is completely detubularized for neobladder formation.

**Figure 17:**
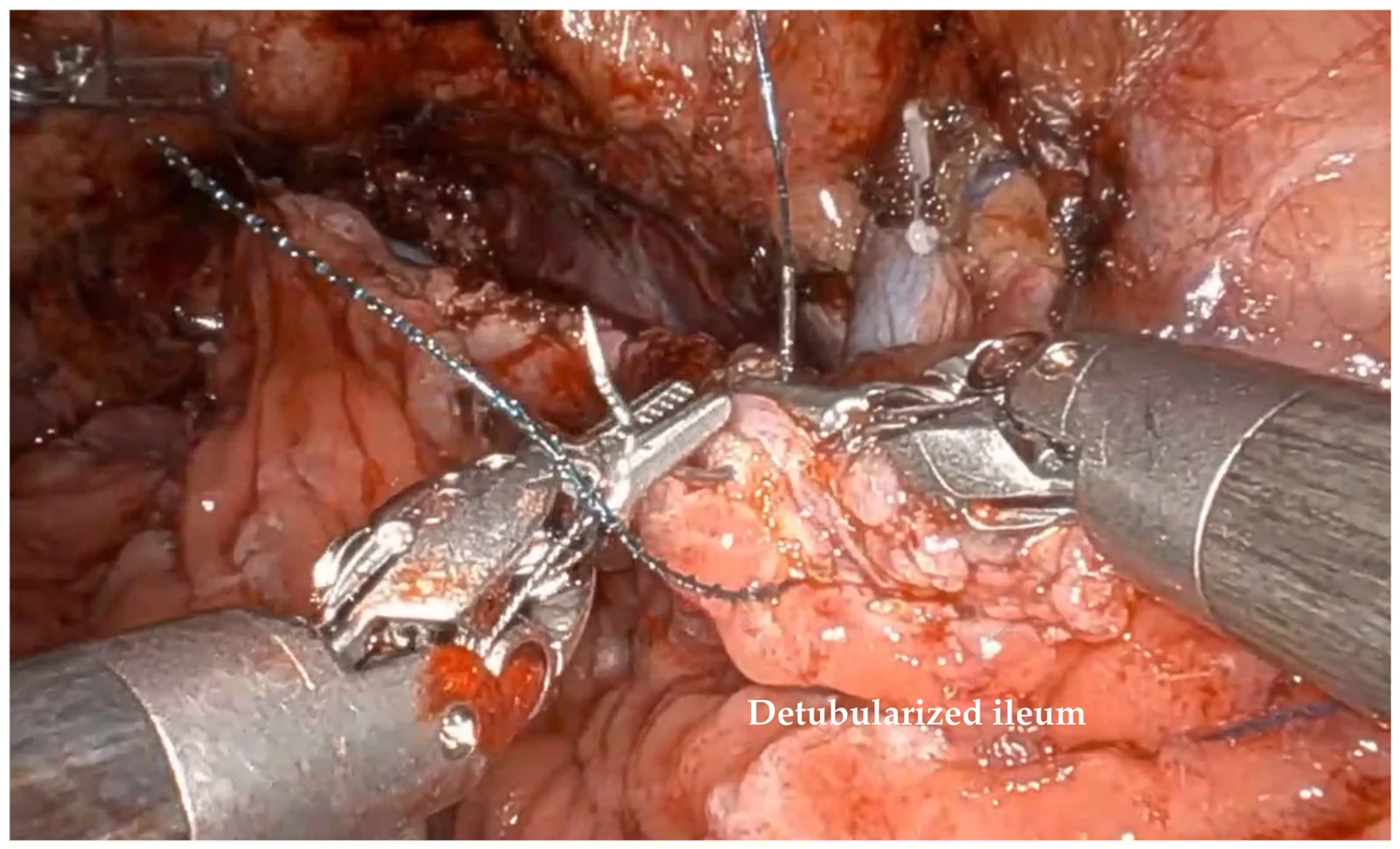
Suturing of the posterior neobladder plate. A stay suture is used to stabilize the bowel and facilitate suturing.

The bowel segment is detubularized along its antimesenteric border using monopolar scissors, with the exception of the proximal 10 cm, which is reserved for the afferent limb (Figure 15 and Figure 16). We favor the modified Studer neobladder initially described by Wiklund et al. [36]. Three stay sutures are placed to form an inverted ‘L’-shaped configuration. The posterior neobladder plate is closed using multiple 6-inch 3-0 barbed sutures (Figure 17). This approach reduces funneling of the neobladder toward the urethra and allows a spherical configuration that, on theoretical grounds, minimizes intraluminal pressure and may thereby aid continence. Closure of the anterior neobladder is begun with a stay stitch that folds the detubularized segment toward the distal cut edge of the afferent limb, and the edge toward the urethro-ileal anastomosis is closed in a continuous fashion using barbed sutures.

The uretero-ileal anastomoses to the afferent limb are then completed as described in Section 4.4, with a 6 Fr single-J stent passed through each anastomosis and out through the opening of the anterior neobladder. Finally, the remaining portion of the anterior neobladder is closed using 3-0 barbed sutures, and the distal ends of the single-J stents are brought up to the skin through the anterior abdominal wall using an artery forceps. Diverting urine through the stents is intended to reduce the risk of urinary leak, which may contribute to postoperative ileus.

A leak test with 120 mL of saline is performed, and any defects are oversewn. A 19 Fr JP drain is then placed in the pelvis.

## 5. Postoperative Care and Enhanced Recovery

Enhanced recovery is the standard of care after radical cystectomy, and the European Association of Urology Robotic Section has published a consensus to standardize postoperative care [22]. Much of the underlying evidence derives from colorectal surgery, where enhanced recovery reduces complications and length of stay without compromising safety [23]. The components described below reflect our institutional pathway; elements supported by randomized evidence are distinguished from those reflecting local practice, for which considerable variation exists between centers (Table 1).

### 5.1. Analgesia, Prokinetics, and Thromboprophylaxis

In our experience, intrathecal morphine and quadratus lumborum blocks provide effective analgesia and minimize opioid requirements. Where available, patients receive alvimopan, a peripherally acting mu-opioid receptor antagonist, 12 mg twice daily, which has been shown to shorten the time to recovery of bowel function after radical cystectomy [19]; this is commenced before surgery and continued for up to five days. In our practice, intravenous metoclopramide 10 mg three times daily is also prescribed as a prokinetic, although evidence for its effect on postoperative ileus is limited. All patients receive intravenous acetaminophen until bowel function recovers, after which it is converted to the oral form. Adjuncts such as lidocaine patches and gabapentin may provide additional non-opioid analgesia, and where renal function is normal, a nonsteroidal agent such as ketorolac 30 mg up to four times daily is helpful in minimizing opioid use. An H_2_-receptor antagonist is prescribed for gastroprotection as part of our local protocol. Extended pharmacological thromboprophylaxis for approximately four weeks after cystectomy is supported by observational data showing a reduction in post-discharge venous thromboembolism [26]. In our practice, subcutaneous heparin is used until renal function is confirmed normal, after which it is switched to low-molecular-weight heparin; at discharge, this is converted to a direct oral anticoagulant to complete a 30-day course, an off-label approach supported by observational series rather than randomized data [27].

### 5.2. Early Feeding and Mobilization

Patients are encouraged to sit out of bed in a chair for four hours on the day of surgery and to mobilize shortly thereafter, aiming to walk at least five times daily, to use an incentive spirometer to prevent atelectasis, and to perform limb exercises. A nasogastric tube is not required postoperatively; the tube placed at induction is removed at the end of the operation. In our pathway, patients begin with sips of fluids and, on the first postoperative day, progress to clear liquids with a 1500 mL fluid restriction to reduce the risk of aspiration. On the second postoperative day, they advance to a full liquid diet. Most patients pass flatus on the night of the second postoperative day, after which they progress to a soft and then a regular diet. Carbonated drinks are avoided to prevent bowel distension. A low-residue diet and an oral laxative such as polyethylene glycol 3350 are used until bowel function has normalized, and simethicone, as required, is helpful for bloating. These specific dietary steps and restrictions reflect local practice; the evidence-based ERAS principle is early resumption of oral intake as tolerated [22].

Neobladder patients are taught to flush the reservoir daily to prevent mucus accumulation.

### 5.3. Drains, Stents, and Discharge

**Figure 18:**
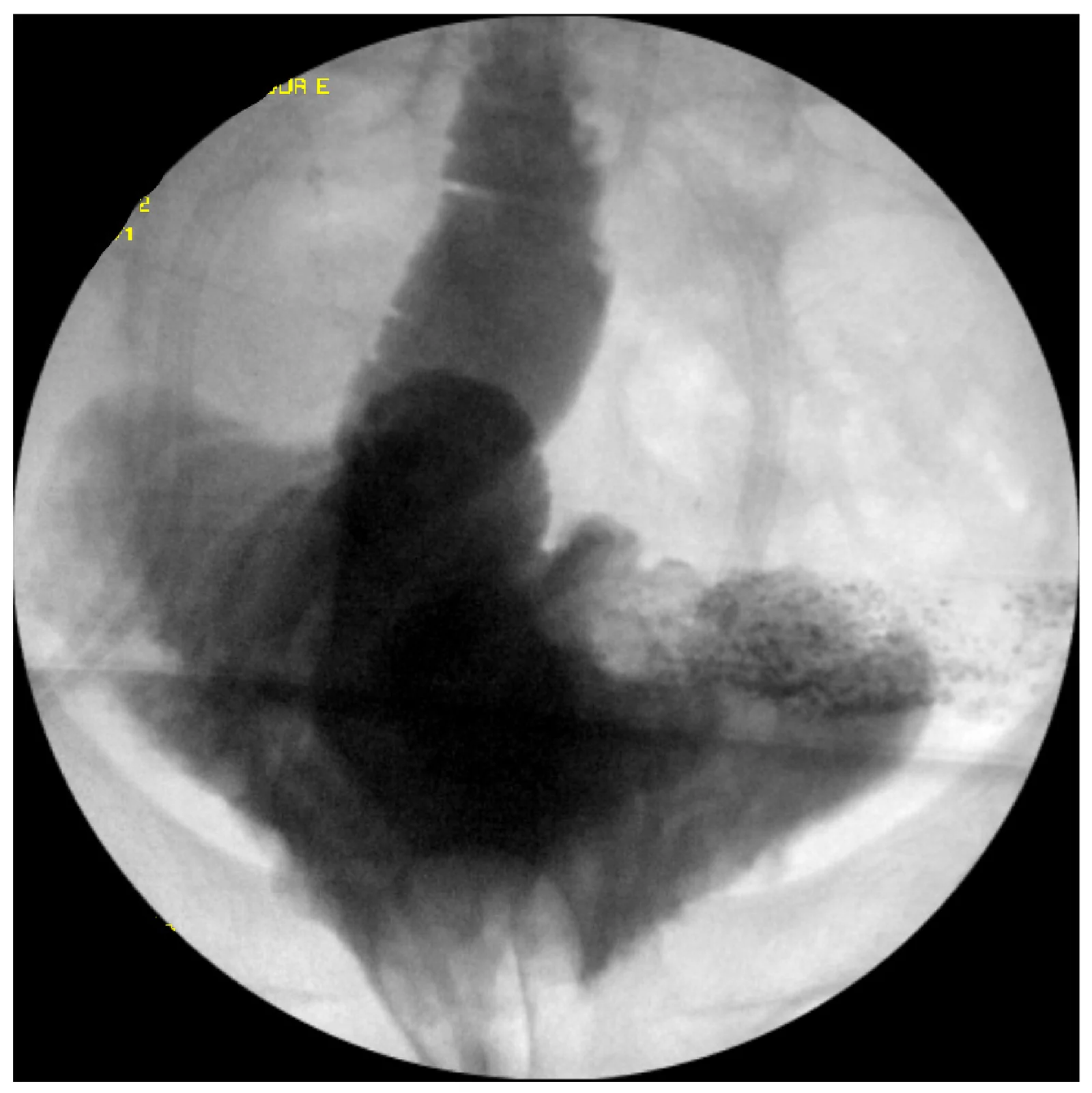
Cystogram view of neobladder.

In our institutional experience, the typical length of stay is approximately four days. The pelvic drain is removed when its output is low and the drain fluid creatinine matches the serum level, confirming the absence of a urinary leak. Ileal conduit stents are removed in clinic 7–10 days postoperatively. In neobladder patients, the ureteric stents and urethral catheter are removed at 21 days following a cystogram that confirms the integrity of the reservoir (Figure 18). In our practice, ciprofloxacin is started the day before stent removal and continued for a total of three days to reduce the risk of sepsis; this is a local protocol, and antibiotic cover for stent removal varies between centers in the absence of comparative evidence.

## 6. Outcomes

### 6.1. Complications

Complications after iRARC are conveniently classified as early or late, and gastrointestinal and infective complications predominate. Postoperative ileus remains the most common reason for prolonged hospital stay, which is the rationale for the opioid-sparing, prokinetic, and goal-directed strategies described above [7]. Surgical complications account for the majority of major 90-day events; in experienced hands, the risk of urinary leak is low and comparable to open surgery, with most leaks managed conservatively by percutaneous drainage or percutaneous nephrostomy [44]. Radical cystectomy demonstrates a well-established relationship between caseload and outcome, such that mortality falls markedly with increasing surgical volume; the considerable cost of acquiring and maintaining a robotic system has, in turn, encouraged a beneficial centralization of cystectomy services [32]. Late complications shared with open diversion include uretero-ileal stricture, urinary tract infection, metabolic acidosis and, in neobladder patients, incomplete emptying; asymptomatic bacteriuria is common and warrants treatment only when symptomatic [45].

### 6.2. Perioperative Outcomes

The strongest evidence for a totally intracorporeal approach comes from the iROC trial, which randomized patients to iRARC or open cystectomy. Patients undergoing iRARC spent more days alive and out of hospital within 90 days (82 vs. 80 days) and experienced fewer thromboembolic complications (1.9% vs. 8.3%) and fewer wound complications (5.6% vs. 16.0%); they also reported better quality of life and less disability at five weeks [7]. Our meta-analysis of randomized trials confirms that RARC is associated with lower blood loss, fewer transfusions, shorter hospital stay, and fewer thromboembolic events than open surgery at the expense of a longer operative time, with no difference in overall complication rates [9]. In a non-randomized institutional comparison, we previously reported that combining iRARC with a comprehensive ERAS program was associated with a shorter length of stay without an increase in 90-day readmission rates [8].

### 6.3. Oncological Outcomes

Randomized data support the oncological non-inferiority of robotic compared with open cystectomy, although the principal trials used extracorporeal diversion. The RAZOR trial, powered for two-year progression-free survival, demonstrated non-inferiority of RARC, with progression-free survival of 72% [4]. The iROC trial, in which recurrence was a secondary end point, found no difference in recurrence rates at a median follow-up of 18 months, and no port-site recurrences were observed in the robotic arms of either trial. A systematic review confirms comparable five-year outcomes, which is reassuring given that fewer than 5% of recurrences occur beyond five years; reported five-year disease-free, recurrence-free, and overall survival following RARC lie in the ranges of 53–74%, 66–80%, and 39–66%, respectively [46]. The histopathological surrogates of oncological adequacy, namely the positive surgical margin rate and lymph node yield, are equivalent between the robotic and open approaches [46].

### 6.4. Functional Outcomes

For appropriately selected patients, nerve-sparing and prostate-capsule-sparing techniques have been associated with better sexual function than standard cystectomy, with at least comparable continence, although the evidence derives largely from non-randomized series [28,29]. Preservation of membranous urethral length and the apical sphincteric complex is considered an important technical determinant of continence after orthotopic reconstruction; however, this is extrapolated from radical prostatectomy data [33] and has not been validated to the same extent in patients undergoing cystectomy. Daytime continence rates after intracorporeal neobladder are high, and supporting the reservoir with anterior and lateral sutures may reduce the risk of retention from kinking at the neobladder–urethral junction [47].

### 6.5. Conclusions

Robot-assisted radical cystectomy with intracorporeal urinary diversion is a safe and feasible option in experienced, high-volume centers. Randomized data report a modest increase in days alive and out of hospital and reduced thromboembolic and wound complications versus open surgery; recurrence, a secondary end point, was also similar. Observational data suggest that patients with limited physiological reserve may derive particular benefit, but this requires prospective confirmation. The technique described here reflects the experience of a single high-volume institution, and several perioperative elements represent local practice rather than evidence-based requirements. Future studies should define which patients benefit most from iRARC and how surgery is best integrated with rapidly evolving perioperative systemic therapy.

## Figures and Tables

**Table 1:** Evidence base for the principal elements of the iRARC pathway described in this review.

Element	Basis of Evidence	References
***Randomized evidence***		
iRARC vs. ORC: more days alive and out of hospital at 90 days; fewer thromboembolic and wound complications	RCT; meta-analysis of RCTs	[7,9]
RARC vs. ORC: oncological non-inferiority (extracorporeal diversion)	RCT; meta-analysis of RCTs	[4,9]
Standard rather than extended pelvic lymph node dissection	Two RCTs	[10,11]
Perioperative systemic therapy selected according to cisplatin eligibility	RCTs; individual patient data meta-analysis	[12,13,14,15,16,17,18]
Alvimopan to accelerate gastrointestinal recovery	RCT	[19]
Antibiotic prophylaxis limited to 24 h	RCT (open cystectomy)	[20]
Multimodal prehabilitation	RCT (improves functional capacity; effect on complications uncertain)	[21]
***Observational evidence and consensus***		
Enhanced recovery pathway after cystectomy	Consensus statement; colorectal RCTs; institutional cohort	[8,22,23]
Perioperative benefit of iRARC in patients with low cardiorespiratory fitness or anemia	Institutional cohort studies	[24,25]
Extended (30 day) pharmacological thromboprophylaxis; oral anticoagulant as an alternative	Observational cohorts	[26,27]
Nerve-, capsule-, and prostate-sparing cystectomy in men	Systematic reviews of non-randomized studies	[28,29]
Pelvic organ-preserving cystectomy in women	Systematic review of non-randomized studies	[30]
Learning curve of 20–30 cases	Expert consensus	[31]
Hospital and surgeon volume	Population-based cohort	[32]
***Extrapolated from other procedures***		
Membranous urethral length and continence	Radical prostatectomy (meta-analysis)	[33]
Energy-based vs. athermal neurovascular bundle dissection	Radical prostatectomy (non-randomized)	[34]
Physiological tolerance of steep Trendelenburg positioning	Robotic prostatectomy (physiological study)	[35]
***Institutional preference and technical expert opinion***		
Spinal morphine with bilateral quadratus lumborum blocks and local anesthetic adjuvants	Institutional protocol	–
48-h antibiotic prophylaxis; ciprofloxacin around stent removal	Institutional protocol	–
Routine metoclopramide, H_2_-receptor antagonist, and staged dietary progression	Institutional protocol	–
Port configuration, instrumentation, and stapled bowel isolation	Technical preference	–
Neobladder configuration; stent and catheter durations	Technical preference	[36,37]

iRARC, robot-assisted radical cystectomy with totally intracorporeal urinary diversion; ORC, open radical cystectomy; RARC, robot-assisted radical cystectomy; RCT, randomized controlled trial.

## Data Availability

No new data were created or analyzed in this study. Data sharing is not applicable to this article.
